# Unravelling the Strain: Accurate Ring Strain Energies in Chalcogeniranes and Predictive Models for Most p‑Block Three-Membered Rings

**DOI:** 10.1021/acs.inorgchem.5c01736

**Published:** 2025-06-03

**Authors:** Arturo Espinosa Ferao

**Affiliations:** † Depto. Química Orgánica, Facultad de Química, Campus de Espinardo, 16751 Universidad de Murcia, 30100 Murcia, Spain

## Abstract

State-of-the-art single reference computational methods were utilized to accurately determine the ring strain energy (RSE) for all three-membered rings (3MRs) containing the *El*_2_ *Ch* core, where *Ch* is a chalcogen atom and *El* is any element of groups 13 to 16, from the second to the sixth period. Only thallium was excluded as *El* element because *Tl*_2_ *Ch* energy minima were demonstrated to constitute pseudorings (absence of ring critical point). The p-character of the atomic orbitals employed in endocyclic bonding, the destabilizing repulsion of electron clouds in chalcogen-chalcogen bonds in trichalcogeniranes and the existence of 2π-electron Hückel-type aromaticity in Tr_2_ *Ch* rings were identified as key electronic factors influencing RSE. Finally, the increased number of 3MRs for which accurate RSE has been reported so far allows widening the additive methodology for the estimation of RSE to an extended set of one hundred and sixty-one endocyclic bonds with remarkable accuracy (root-mean-square error 1.21 kcal/mol).

## Graphical abstract

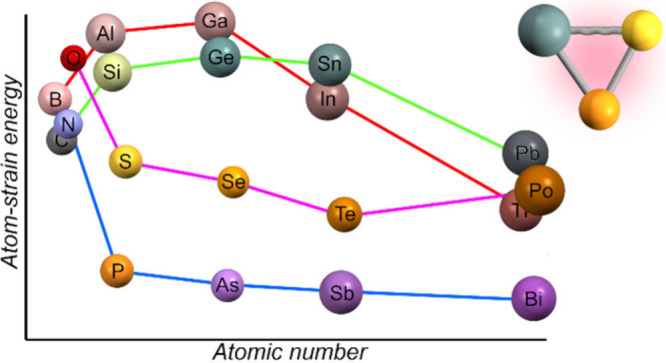

## Introduction

Three-membered rings (3MRs) composed of p-block elements from groups 13 to 16 have long captured the attention of chemists due to their high ring strain energies (RSE), which drive their unique reactivity and synthetic utility. These properties have made such systems pivotal in understanding the interplay between structure and stability in strained small-ring systems,^1–3^ and their high reactivity underpins their utility in synthetic and materials chemistry. Among these, heterocycles such as oxiranes^4^ serve as key intermediates, widely used in ring-opening polymerization (ROP) to produce polyethers, with diverse industrial applications, and other molecular transformations^5^ due to their polar C–O bonds and high RSE.

Other strained heavier chalcogen- and pnictogen-containing rings^6^ hold potential for designing new materials with tailored properties.^2^ Additionally, strained 3MRs often serve as reactive intermediates, enabling access to functionalized organic and inorganic compounds that would otherwise be difficult to synthesize.^6^ On the other hand, emerging classes like dichalcogeniranes open avenues for further exploration of strain and bonding in lighter and heavier group elements, respectively.^2,7^ The design of dioxiranes, for example, has revolutionized oxidation chemistry due to their unique reactivity,^8^ whereas oxathiirane is only known at low temperature in argon matrices.^9^ The fate of other heterodioxiranes *ElO*_2_ (*El* = Si, Ge, Sn, N, P, Sb, S, Se) has also been reviewed.^10^ The broader family of dichalcogeniranes, *ElCh*_2_, characterized by two identical chalcogen atoms (*Ch*) and an additional p-block element (*El*) from the second to the sixth row, represent a largely unexplored class of 3MRs. These systems are of particular interest because they could combine potentially high strains with heavy-element effects, such as relativistic stabilization and unique bonding interactions. Such heavier dichalcogeniranes are now being explored for their potential to stabilize heavy-element bonding motifs and facilitate novel ring expansion reactions.^7^ Recent advances in the synthesis and characterization of diseleniranes and ditelluriranes have revealed unprecedented bonding features, including long chalcogen-chalcogen single bonds and novel donor–acceptor interactions.^7^ These findings not only extend our understanding of strain effects in main group chemistry, but also highlight the untapped potential of such systems for materials design and catalysis. Reported dichalcogeniranes containing other p-block element with its typical (lowest) covalency include dioxaphosphirane (named as *PO*_2_ using only the ring containing atoms),^11^ diselenasiliranes (*SiSe*_2_),^12^ -stanniranes (*SnSe*_2_)^13^ and -phosphiranes (*PSe*_2_).^14^ However, there is a significant gap in the available RSE data for these systems, which could provide crucial insight into the interplay between strain and electronic structure, particularly for heavier chalcogens and p-block elements, where these effects are amplified.

The study of RSE in small-sized rings is therefore of great importance in both theoretical and practical chemistry. From a theoretical perspective, RSE quantifies the energetic penalty imposed by geometric constraints in rings, providing a fundamental understanding of strain effects and their correlation with reactivity. High RSE values in 3MRs are a direct consequence of deviations from ideal bond angles and torsional strain, providing a driving force for ring opening, rearrangement and polymerization reactions.^3,15^ These properties make 3MRs ideal models for investigating concepts such as hybridization strain, lone-pair interactions, and the influence of electronegativity or aromaticity on reactivity.^16^ The inclusion of unsaturated 3MRs further broadens this understanding, highlighting how the introduction of π bonds increases strain, while occasionally introducing stabilizing factors such as aromaticity, which decreases as the group descends.^16^

Furthermore, the deployment of strained three-membered rings as latent scaffolds for frustrated Lewis pair (FLP) chemistry represents an intriguing frontier in synthetic organic chemistry, due to the resultant ability to modulate electronic characteristics upon activation. The concept of leveraging such strained systems as precursors or ’masked’ versions of FLPs has been underexplored but possesses substantial potential for novel bond activation and catalysis strategies. Notably, Stephan and Erker demonstrated that cyclic (alkyl)­(amino)­carbenes, which can be considered as cyclic analogs to traditional FLPs, exhibit reactivity reminiscent of bona fide FLPs due to the presence of adjacent Lewis acidic and basic sites.^17^ The application of small, strained rings could extend this paradigm by temporarily masking one or both of the Lewis sites, as reported for the P/N frustrated Lewis pair in superstrained 3-imino-azaphosphiridine complexes.^18^ This approach could pave the way for developing new methodologies in catalysis, particularly in transformations where controlled reactivity (activation) and the stabilization of high-energy intermediates are crucial.

By systematically calculating RSE for these systems, it becomes possible to predict and exploit their reactivity in targeted synthetic strategies. Previous studies have highlighted the importance of accurately calculating RSE using homodesmotic reactions to quantify the inherent strain in both saturated^1^ and unsaturated^15^ 3MRs. While such accurate methods provide benchmarks essential for theoretical development, their computational cost necessitates the development of faster, more generalizable additive approaches. These methods, based on atom- or bond-specific contributions, offer a practical compromise, allowing efficient estimation of RSE for a wide range of systems while maintaining acceptable accuracy.^2,5^ For instance, the use of bond strain contributions in additive models allows RSE predictions with reduced error margins compared to previous methods, with sufficient accuracy for applications ranging from materials science to reactive intermediates.^10^ It is important to note that, thus far, the additive methodology is based on the hitherto reported set of 108 bond-strain parameters,^5^ which enables the rapid and dependable estimation of RSE for 771 rings comprising solely of El-El, El-Tt (Tt = tetrel) and El-Pn (Pn = pnictogen) bonds. This figure represents 80.0% of the maximum number of 969 possible 3MRs that could be formed using the set of 17 group 13–16 elements from the second to the sixth row (with the exception of the most metallic elements In, Th and Pb, which often do not form proper three-membered cyclic structures,^1^ from the total 20 elements).

Leaving aside bonds involving triels, which only in very isolated cases form part of regular three-membered rings,^2,19^ El-Ch bonds constitute the important missing piece to be able to extend the additive method of RSE estimation by bond strain contributions to practically all 3MRs containing any p-block element. Hence, the results of this study on RSE of symmetric chalcogenadielementiranes, *X_2_Ch* (Figure 1), which are presented here, contribute to a comprehensive understanding of RSE. Here, Ch represents any chalcogen atom and X is an element of groups 13–16 with its most characteristic covalence (3, 4, 3, and 2 for groups 13 to 16, respectively) completed by bonds to H, and named as **1** ^**El**^_**Ch**_ (“El” referring to the heavy element of the X moiety) or, by making use of only ring atoms, as *El*_2_ *Ch*. This is achieved by integrating high-accuracy benchmark data with a generalized and refined additive model. The latter will provide a predictive framework for designing and analyzing strained ring systems across the periodic table. This will serve to bridge critical gaps in the existing literature and establish a robust foundation for future explorations into the intriguing reactivity and applications of strained 3MRs. It is anticipated that this will pave the way for innovations in synthesis and materials chemistry.

**Figure 1:**
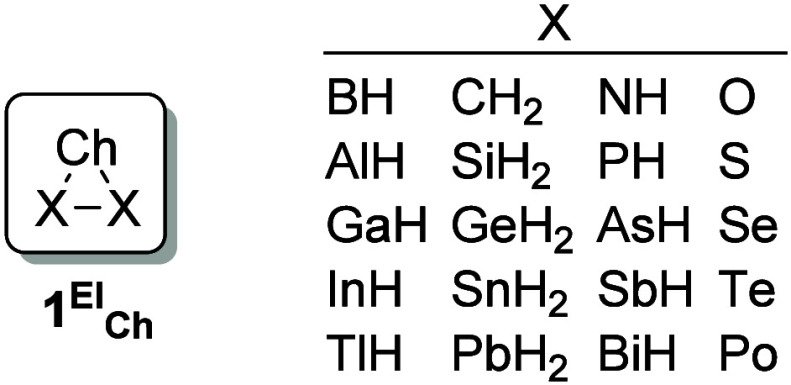
Saturated three-membered heterocycles **1** ^**El**^_**Ch**_ studied (Ch = chalcogen atom).

## Results and Discussion

### A. Accurate Ring Strain Energies – Scope and Limitations

For all 3MRs herein investigated, their RSEs were obtained using appropriate homodesmotic reactions (reaction class 4, or ″RC4″). According to a recent classification of the reaction types used in thermochemistry, homodesmotic reactions are the second-best type in a hierarchy of increasingly accurate processes.^20^ The RSE was obtained as the opposite of the energy balance (including zero-point energy correction) for the two different bond-cleavage reactions in each case (Scheme 1a), at the DLPNO–CCSD­(T)/def2-QZVPP­(ecp)//B3LYP-D4/def2-TZVP­(ecp) level (see computational details). The two RSE values (RSE1 and RSE2) show rather low dispersion with differences most often below 1 kcal/mol. In cases where the difference exceeded 2.5 kcal/mol, a third hyperhomodesmotic reaction (“RC5”) was checked (Scheme 1b), assuming negligible ring strain for the six-membered ring, and the two closest values were averaged to obtain the final RSE.

**Scheme 1:**
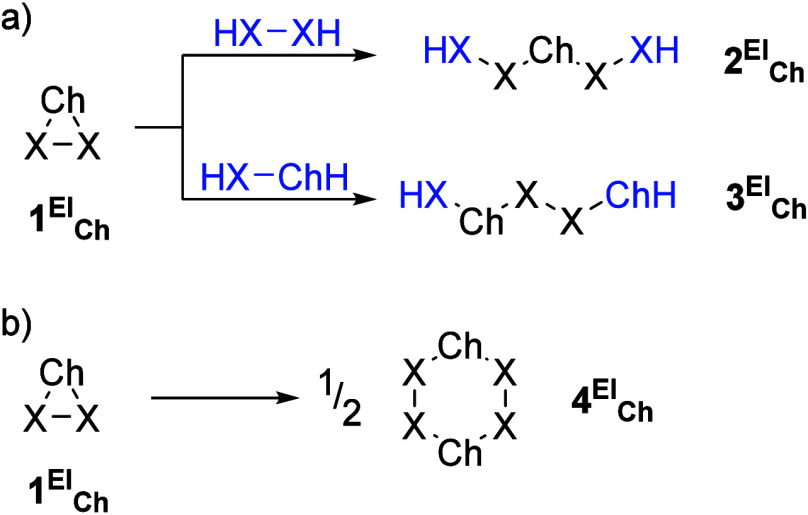
Homodesmotic (a) and Hyperhomodesmotic (b) Reactions Used To Compute RSEs

With a few exceptions, the two energy values thus obtained demonstrated low variability, with a median of 0.43 kcal/mol and a standard deviation of 0.68 kcal/mol (Figure S1). Absolute deviations greater than 2.0 kcal/mol were only observed for *O*_2_ *Te* (3.57) *O*_2_ *Se* (2.60), *Sb*_2_ *O* (2.39), *O*_2_ *Po* (2.24) and *N*_2_ *O* (2.04). The final RSE values are collected in Table 1. It is important to note that, for computationally estimated energies expressed in kcal/mol, only the first decimal place should be considered. However, it should be mentioned that the tabulated values include a second decimal place for the purpose of further mathematical treatment in the estimation of additive contributions to the ring strain (see section D).

**Table 1:** Computed [DLPNO–CCSD­(T)/def2-QZVPP­(ecp)//B3LYP-D4/def2-TZVP­(ecp)] Ring Strain Energies (kcal/mol) for Compounds **1** ^**El**^_**Ch**_ (Named by the Three Ring Atoms)

Group 13		Group 14		Group 15		Group 16	
*B*_2_ *O*	31.40	*C*_2_ *O*	26.13^a^	*N*_2_ *O*	19.43	*O*_3_	29.49^f^
*B*_2_ *S*	20.80	*C*_2_ *S*	17.66^b^	*N*_2_ *S*	27.02	*O*_2_ *S*	35.63
*B*_2_ *Se*	19.91	*C*_2_ *Se*	16.51^c^	*N*_2_ *Se*	24.30	*O*_2_ *Se*	34.92
*B*_2_ *Te*	19.00	*C*_2_ *Te*	15.08^d^	*N*_2_ *Te*	22.48	*O*_2_ *Te*	34.28
*B*_2_ *Po*	17.37	*C*_2_ *Po*	12.09^e^	*N*_2_ *Po*	17.59	*O*_2_ *Po*	27.64
							
*Al*_2_ *O*	45.86	*Si*_2_ *O*	46.23	*P*_2_ *O*	21.64	*S*_2_ *O*	40.51
*Al*_2_ *S*	28.50	*Si*_2_ *S*	24.97^k^	*P*_2_ *S*	10.13	*S*_3_	30.07^g^
*Al*_2_ *Se*	26.67	*Si*_2_ *Se*	23.51	*P*_2_ *Se*	9.52	*S*_2_ *Se*	28.18
*Al*_2_ *Te*	23.90	*Si*_2_ *Te*	21.63	*P*_2_ *Te*	9.67	*S*_2_ *Te*	26.98
*Al*_2_ *Po*	22.69	*Si*_2_ *Po*	20.39	*P*_2_ *Po*	9.16	*S*_2_ *Po*	27.30
							
*Ga*_2_ *O*	41.32	*Ge*_2_ *O*	42.19	*As*_2_ *O*	20.49	*Se*_2_ *O*	36.58
*Ga*_2_ *S*	29.96	*Ge*_2_ *S*	29.99	*As*_2_ *S*	10.23	*Se*_2_ *S*	26.91
*Ga*_2_ *Se*	28.35	*Ge*_2_ *Se*	25.73	*As*_2_ *Se*	9.30	*Se*_3_	26.22^h^
*Ga*_2_ *Te*	25.79	*Ge*_2_ *Te*	23.90	*As*_2_ *Te*	9.36	*Se*_2_ *Te*	25.12
*Ga*_2_ *Po*	24.42	*Ge*_2_ *Po*	22.54	*As*_2_ *Po*	9.01	*Se*_2_ *Po*	24.37
							
*In*_2_ *O*	38.27	*Sn*_2_ *O*	40.97	*Sb*_2_ *O*	23.30	*Te*_2_ *O*	37.32
*In*_2_ *S*	30.02	*Sn*_2_ *S*	28.40	*Sb*_2_ *S*	10.79	*Te*_2_ *S*	25.21
*In*_2_ *Se*	28.61	*Sn*_2_ *Se*	26.96	*Sb*_2_ *Se*	10.50	*Te*_2_ *Se*	24.03
*In*_2_ *Te*	26.81	*Sn*_2_ *Te*	26.07	*Sb*_2_ *Te*	10.37	*Te*_3_	23.25^i^
*In*_2_ *Po*	25.51	*Sn*_2_ *Po*	24.96	*Sb*_2_ *Po*	9.91	*Te*_2_ *Po*	22.78
							
		*Pb*_2_ *O*	36.28	*Bi*_2_ *O*	21.16	*Po*_2_ *O*	33.81
		*Pb*_2_ *S*	28.30	*Bi*_2_ *S*	10.34	*Po*_2_ *S*	23.87
		*Pb*_2_ *Se*	27.12	*Bi*_2_ *Se*	10.11	*Po*_2_ *Se*	22.69
		*Pb*_2_ *Te*	25.39	*Bi*_2_ *Te*	9.97	*Po*_2_ *Te*	21.69
		*Pb*_2_ *Po*	24.42	*Bi*_2_ *Po*	9.74	*Po*_3_	21.53^j^

a 27.86 kcal/mol in ref 2.

b 20.38 kcal/mol in ref 2.

c 18.23 kcal/mol in ref 2.

d 16.23 kcal/mol in ref 2.

e 12.55 kcal/mol in ref 2.

f 28.42 kcal/mol in ref 3.

g 28.77 kcal/mol in ref 3.

h 25.51 kcal/mol in ref 3.

i 26.52 kcal/mol in ref 3.

j 24.98 kcal/mol in ref 3.

k 24.68 kcal/mol in ref 3.

Chalcogenadithaliranes *Tl*_2_ *Ch* have been observed to not exist as proper cyclic structures. This is due to the absence of a Tl···Tl bond path, as evidenced by Atoms-In-Molecules^21^ (AIM) analyses, which did not reveal any ring critical points (RCPs). In the “*Tl*_2_ *O*” pseudoring **1** ^**Tl**^_**O**_ *, as a case in point, a bond critical point (BCP) and bond path is observed between the O atom of one formal “H–Tl=O” unit and the heavy atom of the remaining “H–Tl”: unit (Figure 2a). The Natural Bond Orbital^22^ (NBO) analysis describes the first unit with a single Tl–O bond (d = 1.914 Å; WBI = 0.964) featuring positive (natural) charge at Tl (q^N^_Tl_ = +1.23 e) and negative charge at O (q^N^_O_ = −1.12 e). Two electron transfers are observed from ‘p’ atomic orbitals (AO) at the O atom (1.857 and 1.831 e) to two formally empty (0.093 and 0.024 e) ‘p’ AO at the other Tl atom, with Second Order Perturbation Theory (SOPT) associated energy of E_SOPT_ = 24.1 kcal/mol. The dative bonding nature of this O→Tl interaction is pointed out by its relative weakness (d = 2.559 Å; WBI = 0.230) and confirmed by the typical diagnostic pattern^23^ showing low positive value of ∇^2^ ρ (3.19 e/Å^5^) and the occurrence of the two valence-shell charge concentration (VSCC) bands falling within the basin of the donor atom (Figure 2b). Evidence for this can be found in the shallow VSCC_Tl_ band minimum, which is located slightly (0.06 Å) to the left of the BCP. This gives rise to a positive value for the *relative charge concentration band position* parameter,^23b^ τ = 0.008, with diagnostic relevance. The Tl···Tl interaction (d = 3.141 Å; WBI = 0.266) is weak and predominantly of electrostatic nature. According to the NBO analysis, this interaction arises from electron donation from a filled (1.878 e) ‘s’ AO in the second Tl atom, to several ‘p’ AOs in the first Tl atom (the four most intense interactions amounting to E_SOPT_ = 18.1 kcal/mol).

**Figure 2:**
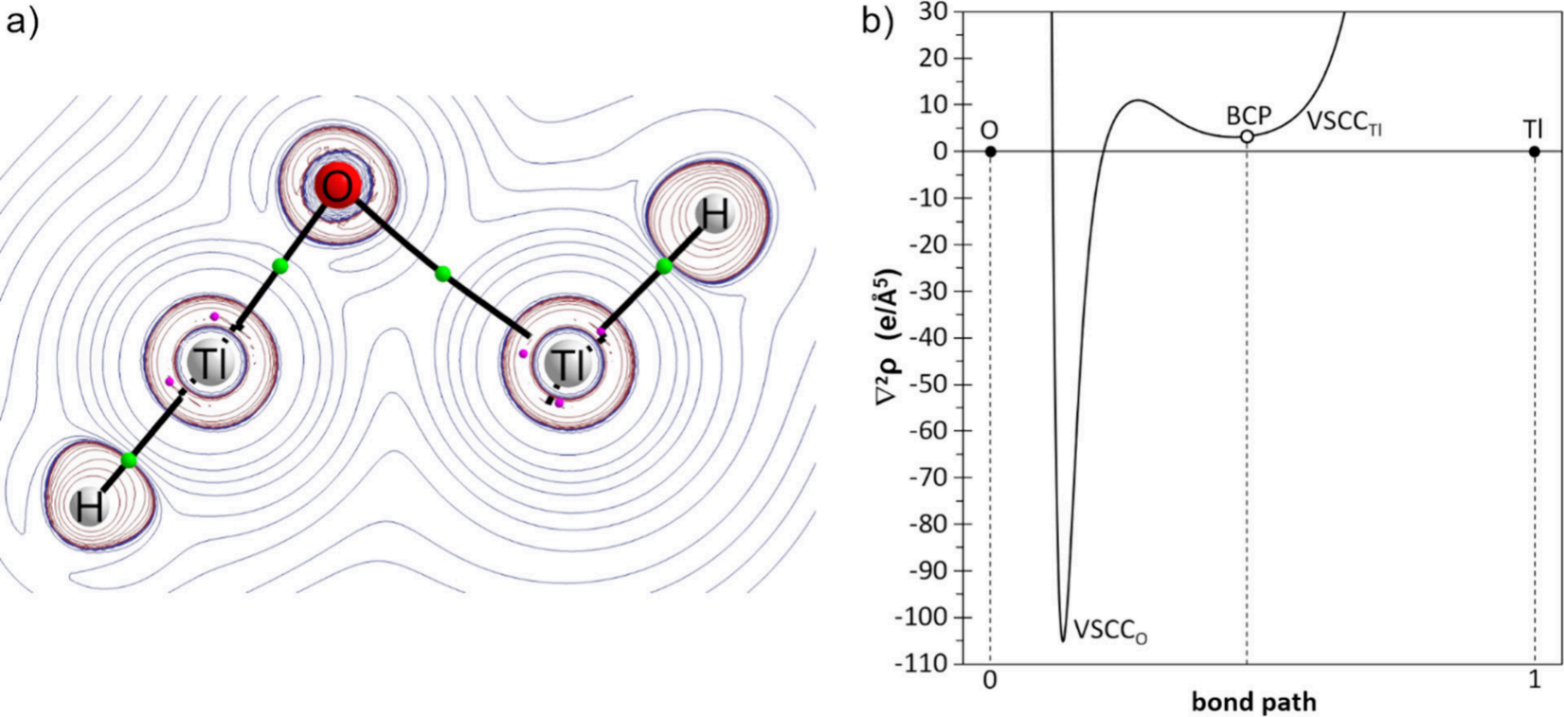
Computed (B3LYP-D4/def2-TZP) contour plot of ∇^2^ ρ in the molecular plane (a) and plot of ∇^2^ ρ along the O–Tl bond path (b) for pseudoring **1** ^**Tl**^_**O**_ *.

With the exception of polonium, the acyclic pseudoring structures **1** ^**Tl**^_**Ch**_ * have the capacity to interconvert to the corresponding degenerated structures (Scheme 2) via a low-lying transition state (ΔG^‡^ = 7.1, 8.1, 8.0, and 8.1 kcal/mol, for Ch = O, S, Se and Te, respectively) with a proper cyclic structure (one RCP and three endocyclic BCPs). For instance, in the case of **1** ^**Tl**^_**O**_ *, the TS features a shorter Tl–Tl bond (d = 2.755 Å; WBI = 0.645; ρ_BCP_ = 0.384 e/Å^3^; ∇^2^ ρ_BCP_ = 1.771 e/Å^5^) and two intermediate Tl–O bonds (d = 2.150 Å; WBI = 0.630; ρ_BCP_ = 0.644 e/Å^3^; ∇^2^ ρ_BCP_ = 8.234 e/Å^5^).

**Scheme 2:**
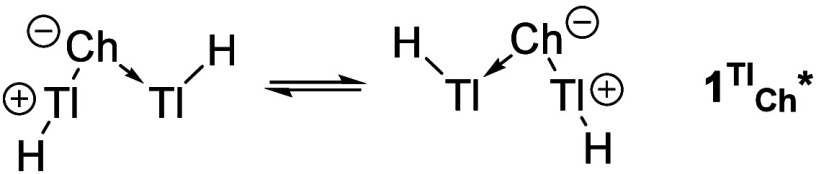
Interconversion of Degenerated Pseudo-Rings **1** ^**Tl**^_**Ch**_ *

For all other proper 3MRs, for any given p-block element ‘El’, the RSE for *El*_2_ *Ch* rings decreases on going down the chalcogen group, except *N_2_O* and *O_3_* that exhibit lower RSE than most heavier *N*_2_ *Ch* and *O*_2_ *Ch* (Ch = S, Se, Te) analogues, respectively (Table 1). Other minor anomalies are also observed for the RSE of *Pn*_2_ *Te* (Pn = P, As) showing slightly higher values than the corresponding *Pn*_2_ *Se* analogues, and the slightly higher value of *S*_2_ *Po* relative to *S*_2_ *Te*. However, the significance of these deviations is deemed negligible, as they fall well within the margin of error inherent to the RSE evaluation method (*vide supra*). When averaging for all five chalcogen elements in *El*_2_ *Ch* rings, the dependency of the RSE on the other p-block element ‘El’ (Figure 3) demonstrates the expected decrease for group 15 and 16 elements on descending the groups, and the higher RSE values for groups 13 and 14, which remain almost invariant except for the lightest elements. In comparison to the additive atom-strain contributions previously reported,^3,6^ the element dependencies presented herein are surprisingly high for chalcogens. Conversely, the elements in the triel group exhibit comparatively low values, particularly for boron. This phenomenon could be tentatively ascribed to the potential stabilization arising from Hückel-type 2π-electron aromaticity *Tr_2_C* *h* rings (*vide infra*).

**Figure 3:**
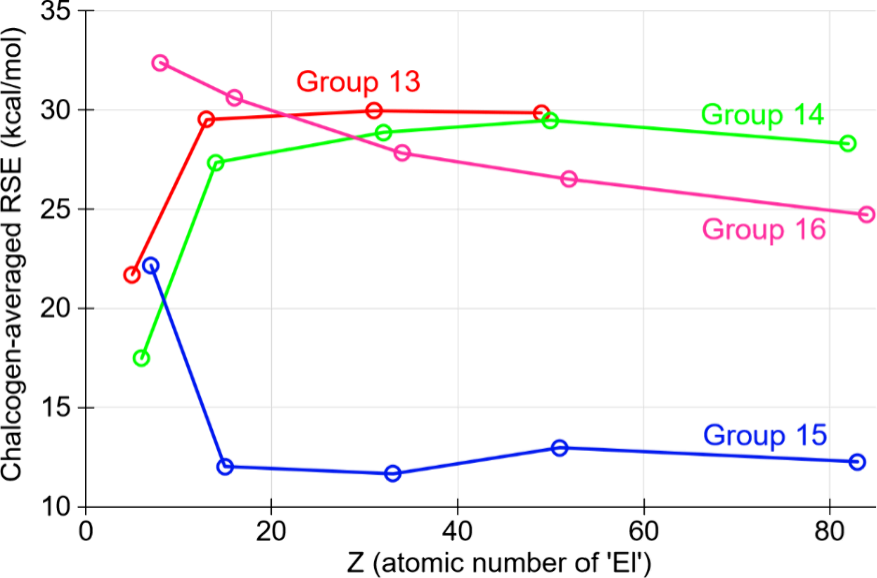
Computed [DLPNO-CCSD­(T)/def2-QZVPP­(ecp)//B3LYP-D4/def2-TZVP­(ecp)] chalcogen-averaged RSE (kcal/mol) for **1** ^**El**^_**Ch**_, plotted against the atomic number of ‘El’.

### B. Effect of Hybridization on RSE

As indicated in earlier reports on other 3MRs, one of the most significant factors influencing RSE is the hybridization of ring atoms. For saturated 3MRs containing solely one ring heteroatom (*C*_2_ *El*), the RSE exhibits a roughly linear correlation with the p-character of the AO that the heteroelement ’El’ utilizes for the endocyclic C-El bonds.^2^ As anticipated, the p-character exhibited by triel and tetrel elements is marginally higher than the 67% and 75% levels observed for purely sp^2^- and sp^3^-hybridized atoms, respectively. This finding provides a compelling explanation for their apparent reluctance to accommodate the relatively modest endocyclic angles in a 3MR, particularly for the heaviest elements that necessitate more acute endocyclic bond angles (Figure 4). In case of pnictogens, only N exhibits a propensity to hybridize. However, the placement of enriched s-character at the lone pair (LP) enables the allocation of enhanced p-character to endocyclic bonds, thereby exerting a strain-relieving effect. In contrast, all other heavier pnictogens utilize a predominantly p-AO configuration for endocyclic bonds, thereby reducing the strain imposed by the ring. A priori, the same effect should be observed for chalcogens, and, indeed, their increased p-character on descending the group (beyond 92%) parallels a decrease in RSE (Figure 4). However, all *Ch’*_2_ *Ch* rings exhibit an additional RSE enhancement that can be tentatively explained by the weakness of chalcogen-chalcogen bonds. This is attributable to the six LPs being allocated around a small 3MR system, resulting in destabilizing electrostatic repulsion of electron clouds. This repulsion is strogest for the short bond distances in O–O bonds and decreases systematically as the atomic size increases. Conversely, an opposite effect is observed when moving down group 16, as the bonding σ orbitals are more diffuse and less effective at forming strong covalent bonds due to poorer orbital overlap.^24^ Overall, the trend in acyclic C–C bond strength can be summarized as follows: S–S and Se–Se bonds are the strongest in this series, with bond strength decreasing for Te–Te and Po–Po, with O–O bond strength being anomalously low, as exemplified in peroxides. The O–O bond in hydrogen peroxide is relatively weak (ca. 51 kcal/mol) in comparison to typical O–H or O–C bonds (approximately 110 and 86 kcal/mol, respectively), and even smaller in simple (dialkyl) peroxides (ca. 34 kcal/mol).^25^ This leads to the facile homolytic cleavage of the peroxide bond, facilitating radical formation and oxidative reactions.^26^ Disulfide bonds (S–S) exhibit bond energies^25b^ of approximately 57 kcal/mol (computed range for diorganyl disulfides 49–69 kcal/mol),^27^ which are notably weaker than corresponding single bonds involving carbon or hydrogen. Disulfide bonds are therefore readily susceptible to homolytic cleavage under moderate conditions, thus rendering them effective radical initiators or susceptible to reduction.^28^ Selenium–selenium bonds in compounds such as diphenyl diselenide have slightly lower dissociation energies^25b^ of around 41 kcal/mol (32–50 kcal/mol).^29^ Such weakness in the bonds promotes facile cleavage under mild reducing conditions or exposure to heat or light.^30^ Ditellurides (Te–Te) possess even weaker bonds (approximately 30 kcal/mol),^25b^ contributing to their chemical instability and sensitivity to oxidation and disproportionation.^31^ A recently reported machine learning approach allows for the prediction of the bond dissociation energy (in kcal/mol) for the Ch-Ch bonds in R-Ch-Ch-R (R = Me/Ph), which deviates slightly from the previously mentioned trend: Se (60.8/53.7) ≥ S (57.1/51.2) > Te (49.5/33.2) ≈ Po (47.6/37.8) > O (39.9/24.9).^32^ Compared to acyclic species, the different and regularly decreasing instability of C–C bonds in trichalcogenirane compounds (decreasing RSE) as one moves down the group (O > S ≫ Se > Te > Po) can be rationalized as follows. Trioxirane *O*_*3*_ is highly unstable due to maximum lone-pair repulsion, extreme angle strain and the loss of a strong π bond (O=O), compared to the acyclic ozone isomer, which has minimal stabilizing effects. Similarly, *S*_*3*_ is highly energetic for comparable reasons, albeit somewhat mitigated by sulfur’s lower electronegativity and reduced π-bond energy loss. The heaviest *Se*_*3*_, *Te*_*3*_ and *Po*_*3*_ analogues are progressively less strained as lone-pair repulsions and angle deformation penalties decrease, and the absence of strong double bonds makes the open forms less advantageous.

**Figure 4:**
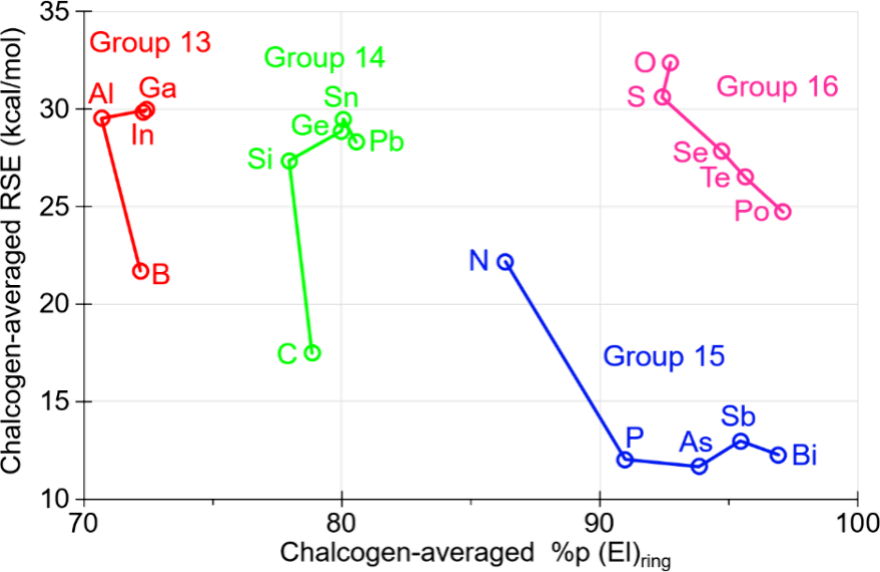
Computed [DLPNO-CCSD­(T)/def2-QZVPP­(ecp)//B3LYP-D4/def2-TZVP­(ecp)] chalcogen-averaged RSE (kcal/mol) versus the p-character (average) of the AO used by ‘El’ in endocyclic bonds of compounds **1** ^**El**^_**Ch**_.

### C. Potential 2π-Electron Aromaticity

As has been previously demonstrated for tripnictogeniranes and trichalcogeniranes,^3^ the occurrence of three ring atoms each bearing (at least) a LP, as in *Pn*_2_ *Ch* and *Ch’*_2_ *Ch*, is not expected to form an aromatic ring sextet. Instead, a remarkable magnetic response can be expected to result from the sum of three atomic diatropic currents.^6^ However, in the case of chalcogenaditrieliranes, *Tr_2_C* *h*, the chalcogen atom can provide a LP allocated in a p-AO (the other LP must be formally allocated in a sp^2^-type AO), which, together with the two vacant p-AOs from the triel atoms, could constitute a Hückel-type 2π-electron aromatic system. Indeed, oxadiborirane *B*_2_ *O* was reported to be quite inert.^33^ Hence, the aromatic character of 15 *Tr_2_C* *h* rings (Tr = B, Al, Ga; Ch = O, S, Se, Te, Po) was examined by employing NICS-related criteria, as recently reported,^6,19^ although many other aromaticity evaluation methods have been proposed.^34^ Benzene was included as a reference, as well as cyclopropenylium cation and 1*H*-borirene as prototypical aromatic 3MRs, and 1*H*-thiirene as representative of antiaromatic 3MRs. Both NICS(0)^35^ and NICS_ZZ_(0) showed too strong a dependence on ring size and only the latter were included in the study. This parameter seems to be misleading as it shows positive values for all *Al*_2_ *Ch* and *Ga*_2_ *Ch* rings, but negative for all other 3MRs including thiirane (Table 2). Total NICS(1)^36^ values (sum of σ+π components) is claimed to represent a rough estimate of the aromatic (diatropic) ring currents, and indicates a high aromatic character for benzene, borirene and cyclopropenylium as well as for all *B*_2_ *Ch* rings, although a clear antiaromaticity is only present for thiirene (Table 2). NICS_ZZ_(1) is often considered more suitable for the study of ring currents,^37^ albeit in the present case it only confirms the same classification of aromatic compounds mentioned above, but does not make a clear distinction between the antiaromatic thiirene and all the other 3MRs.

**Table 2:** Computed [B3LYP/def2-TZVPP­(ecp)//B3LYP-D4/def2-TZVP­(ecp)] Magnetic Aromaticity Related Descriptors for Selected Compounds (π-Only Component in Parentheses)

	NICS_ZZ_(0)^a^	NICS(1)^a^	NICS_ZZ_(1)^a^	NRR	FiPC-NICS_op_ ^a^	∫A·B^z^dz (%)^b^
benzene	–5.50	–10.05 (−14.58)	–10.01 (−11.11)	0.853	–9.99	100.0 (100.0)
borirene	–9.36	–14.68 (−17.26)	–8.85 (−8.20)	0.749	–4.83	39.8 (37.5)
c-C_3_H_3_^+^	–11.01	–14.97 (−13.43)	–9.71 (−3.11)	0.691	–5.46	35.4 (−)^c^
thiirene	–4.77	–2.08 (4.63)	–1.29 (6.38)	0.018	–1.38	14.8 (−35.9)
*B*_2_ *O*	–6.59	–10.83 (−5.05)	–7.15 (−1.41)	0.861	–3.27	22.8 (9.1)
*B*_2_ *S*	–7.98	–14.20 (−8.72)	–8.50 (−2.39)	1.203	–4.27	52.8 (17.1)
*B*_2_ *Se*	–7.15	–15.05 (−9.58)	–8.78 (−2.73)	1.106	–4.84	65.5 (20.5)
*B*_2_ *Te*	–7.56	–16.15 (−11.22)	–9.00 (−3.42)	0.875	–5.18	80.9 (26.9)
*B*_2_ *Po*	–7.87	–16.31 (−11.50)	–8.99 (−3.68)	0.717	–5.22	86.1 (29.8)
*Al*_2_ *O*	0.33	–8.16 (−5.58)	–2.47 (−0.87)	0.002	–1.78	0.0 (7.2)
*Al*_2_ *S*	2.37	–7.67 (−9.64)	–1.57 (−1.78)	0.120	–2.05	16.6 (15.3)
*Al*_2_ *Se*	3.43	–7.82 (−10.47)	–1.43 (−1.94)	0.265	–2.31	25.2 (18.1)
*Al*_2_ *Te*	4.23	–7.77 (−12.25)	–0.96 (−2.21)	0.554	–2.47	34.4 (22.8)
*Al*_2_ *Po*	4.54	–7.77 (−12.76)	–0.80 (−2.32)	0.655	–2.54	38.2 (25.3)
*Ga*_2_ *O*	8.97	–7.15 (−6.25)	–2.01 (−0.99)	0.110	–2.91	24.5 (9.6)
*Ga*_2_ *S*	7.97	–7.11 (−9.24)	–1.60 (−1.78)	0.478	–3.29	42.7 (17.3)
*Ga*_2_ *Se*	8.44	–7.41 (−10.02)	–1.51 (−1.94)	0.616	–3.53	51.1 (20.0)
*Ga*_2_ *Te*	8.70	–7.57 (−11.68)	–1.12 (−2.19)	0.874	–3.64	60.2 (24.3)
*Ga*_2_ *Po*	8.70	–7.60 (−12.24)	–0.97 (−2.29)	0.939	–3.68	63.8 (26.6)

a In ppm.

b Using benzene (100%) and *Al_2_O* (0%) as references for the total function, but only benzene (100%) in the case of the π_ZZ_-only function.

c Not a good fit (R^2^ = 0.295).

Aromatic rings have been shown to exhibit a maximum in their -NICS variation along the *z* axis perpendicular to the mean ring plane,^38^ as observed for all three aromatic reference compounds benzene (black solid line), borirene (blue solid line) and cyclopropenylium cation (purple dashed line) (Figure 5a). Likewise, all *B*_2_ *Ch* and the heaviest *Ga*_2_ *Ch* clearly display a similar aromatic-like pattern, whereas thiirene and *Al*_2_ *O* have a different behavior with the maximum at the origin of the *z* axis (here denoted ‘r’). The NICS rate variation along this *z* axis, defined as [NICS­(r+Δr)-NICS­(r)]/Δr, has been used as a clearer picture for the aromatic or antiaromatic behavior.^19,39^ NICS rate variation plots for aromatic species such as benzene, borirene, cyclopropenylium and all *B*_2_ *Ch* rings start to decrease from zero to a minimum, then increase to a maximum and finally asymptotically decrease toward zero (Figure 5b). This is also the profile shown by the heaviest *Ga*_2_ *Ch* (Ch = S, Se, Te, Po) and, to some extent, the two heaviest *Al*_2_ *Ch* (Ch = Te, Po). Conversely, antiaromatic thiirene (red solid line) and *Al_2_O* (green solid line) start the plot increasing from zero to a remarkably high maximum, then decreasing to negative values (*Al*_2_ *O* crosses to very small negative values at r = 3.86 Å) and finally asymptotically increasing toward zero. Following this criterion, *Al*_2_ *S*, *Al*_2_ *Se* and *Ga*_2_ *O* rings represent borderline cases as they start to decrease to a very shallow minimum (Figure 5b). The ratio (NRR) between the minimum and maximum absolute values has been used as a magnetic aromaticity criterium,^19,39^ according to which all the above-mentioned aromatic rings have NRR > 0.47, the antiaromatic species thiirene, *Al*_2_ *O*, *Ga*_2_ *O* and *Al*_2_ *S* have NRR < 0.13, and *Al*_2_ *Se* remains as a borderline case (Table 2).

**Figure 5:**
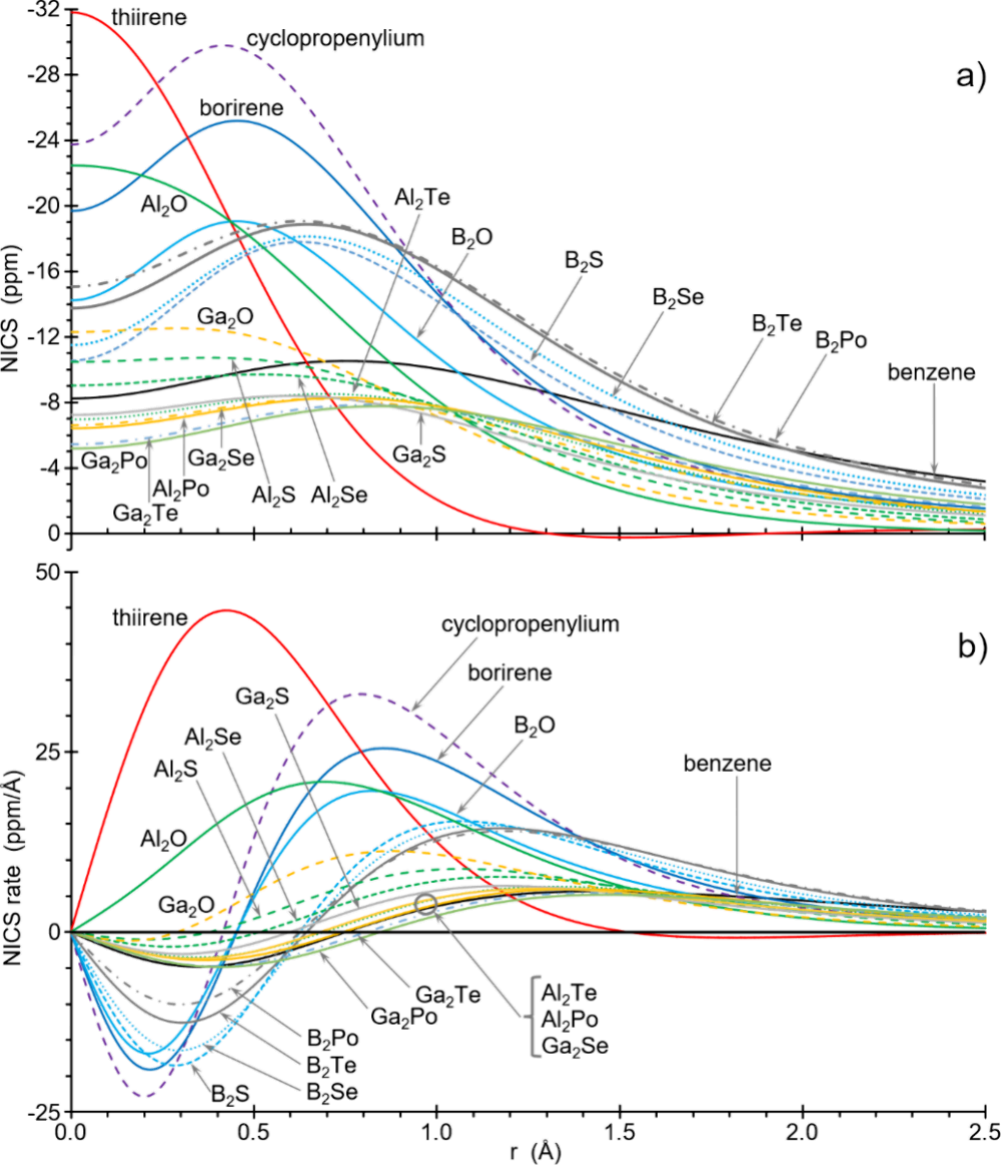
Total NICS (a) and NICS rate (b) variation plots along the perpendicular axis at the ring centroid for selected rings.

An alternative NICS-related approach is the *free of in-plane component NICS* (FiPC-NICS),^19,40^ in which the out-of-plane NICS component (-^1^/_3_(σ_ZZ_)) is plotted versus the in-plane (−^1^/_3_(σ_XX_ + σ_YY_)) NICS component (here referred to as NICS_oop_ and NICS_ip_, respectively) for the set of points along the perpendicular r axis (Figure 6). It is notable that all the most aromatic 3MRs (borirene, cyclopropenylium and all *B*_2_ *Ch*) display the same pattern, with the curve beginning (r = 0.0 Å, at the left-hand side of its plot) with a decrease in both the out-of-plane and in plane NICS components (moving down left in the plot), reaching a first a minimum in NICS_ip_ (left-most edge) and then in NICS_oop_ (bottom edge), from which the NICS_oop_ increases and finally vanishes at long distances (herein computed up to r = 5.0 Å) close to the origin of the Cartesian coordinates (Figure 6). The value of NICS_oop_ at the point at which each curve crosses the vertical NICS_ip_ = 0 axis is defined as FiPC-NICS. The above-mentioned most aromatic3MRs display a substantial negative value of FiPC-NICS, ranging from −3.27 to −5.46 ppm. Notably, benzene demonstrates a significantly higher value of −9.99 ppm (Table 2). Thiirene, conversely, displays a distinct curve shape (Figure 6) and the smallest negative FiPC-NICS value of −1.38 ppm. The remaining 3MRs (*Al*_2_ *Ch* and *Ga*_2_ *Ch*) exhibit a divergent plot pattern, bearing a certain resemblance to that of aromatic compounds, albeit commencing (left-most edge) at the positive NICS_oop_ and negative NICS_ip_ region and progressing through decreasing NICS_oop_ and increasing NICS_ip_ (Figure 6). It is noteworthy that all of them display intermediate FiPC-NICS values within the range of −1.78 to −3.68 ppm, with some of them (*Ga*_2_ *Ch*; Ch = S, Se, Te, Po) falling within the aforementioned range for typical aromatic rings, and *Al*_2_ *O* (or even *Al*_2_ *S*) exhibiting a value very close to that of thiirene (Table 2).

**Figure 6:**
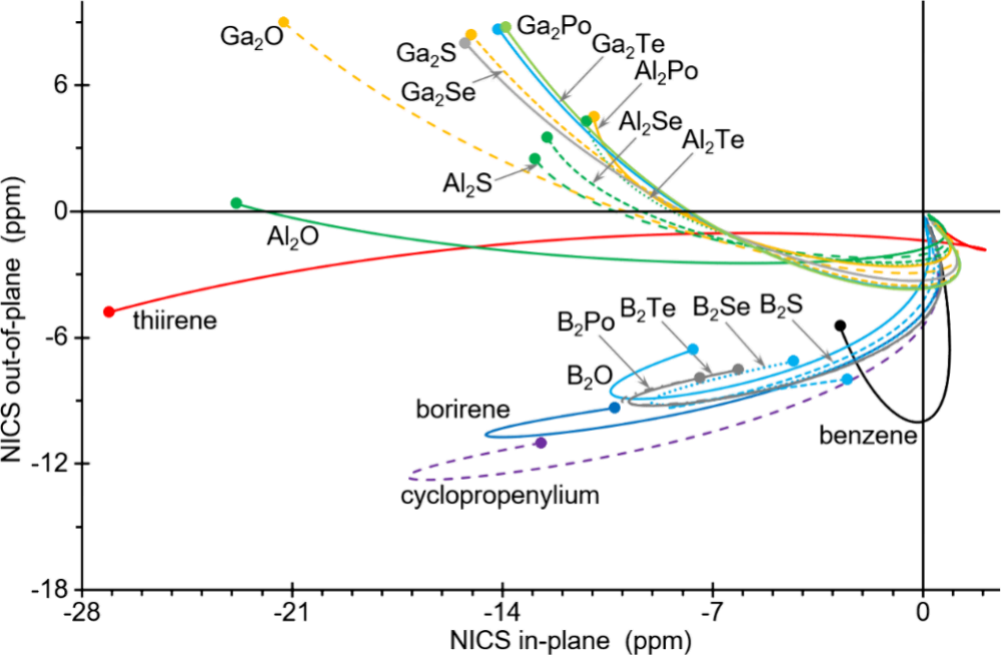
Out-of-plane versus in-plane NICS components variation plot from 0 (small solid circle) to 5 Å distance in the *z* axis perpendicular to the ring plane for selected rings.

Another approach to circumventing the issue of selecting a (arbitrary) distance to quantify NICS-related properties was recently envisaged by Stanger,^41^ who hypothesized that ∫NICS_π,zz_ constitutes a quantitative metric for aromaticity. In order to avoid the use of localized (LMO) or π canonical molecular orbitals (CMO), he proposed the σ-only model (SOM), using the perhydrogenated unsaturated cyclic derivative whose magnetic response is subtracted from the total one (in the non-hydrogenated system) to obtain the π-only quantities. The hydrogenated species were constructed by means of the addition of H atoms to the ring C or Ch atoms, which were displaced from the heavy atoms by the sum of the covalent radii^42^ only in the -z direction.

With this aim the decay region (from 2.5 to 5 Å) of the NICS_π,ZZ_ plots (see Figure S2 for a σ_π,ZZ_ plot) were fit to exponential A·B^r^ functions by a least-squares procedure and the latter integrated between 0 and ∞. Taking the ∞ distance as 100 Å, the integral adopts the operational expression ∫NICS_π,ZZ_ = A·(B^100^ – 1)/ln­(B). This integral is taken as distance-independent aromaticity index and usually given in percentage relative to benzene. For all compounds excepting c-C_3_H_3_^+^, the parameter B ranges within the interval 0.3974–0.5145, although the interval for all *Tr_2_C* *h* rings is much narrower (0.4309–0.4807), and the fitting was remarkably good (R^2^ = 0.9915–0.9976, standard deviation = 0.026–0.062 for all rings). The cyclopropenylium cation was excluded due to its poor regression coefficient (R^2^ = 0.2946). Once more, the obtained values (Table 2) give some aromatic character (>15%) for *Tr_2_C* *h* rings except with the lightest chalcogen and increasing the aromaticity in the series B > Ga > Al and going down the chalcogen group (Figure 7). As reported elsewhere,^6^ aluminum represents an inflection point in the series of the three lightest triels, due to its higher Lewis acidity compared to gallium, despite having similar sizes (r_cov_ = 1.25 Å) due to a “d-block” contraction of the electron cloud in Ga, as a consequence of the lower electronegativity of Al (χ_Al_ = 1.61; χ_Ga_ = 1.81).^43^

**Figure 7:**
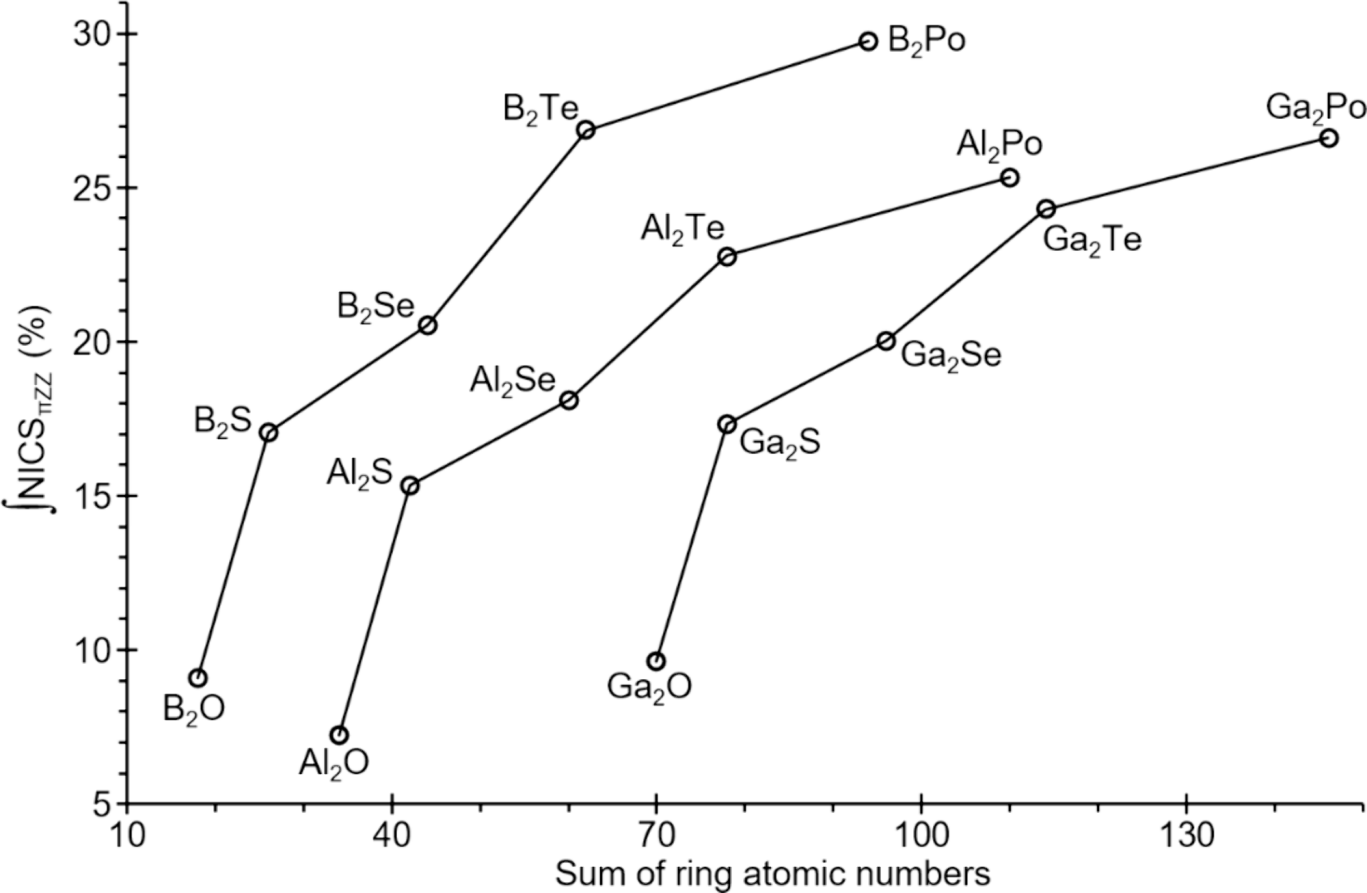
Variation of aromaticity from integration of the NICS_π,ZZ_ function, relative to benzene, for selected rings.

The increase in aromaticity for the Tr_2_ *Ch* ring having heavier chalcogens could be tentatively explained by the lower availability of the LP for the cyclic conjugate system in the lighter chalcogens due to their higher electronegativity. For comparative purposes the same integration was done for the decay approximated function of the total NICS variation curve, in this case using the lowest value obtained for the *Al*_2_ *O* ring as zero, and that for benzene as 100%.

A comparison of the various aromaticity parameters (see Table S1) reveals a number of noteworthy observations. First, there is a strong correlation between NICS_ZZ_(1) and NICS(1) (R^2^ = 0.93398) or NICS_ZZ_(0) (R^2^ = 0.87140). Additionally, there is a significant correlation between the two latter parameters (R^2^ = 0.82137). Some other correlations are worth highlighting. The first of these is the noteworthy linear correlation of ∫NICS_πZZ_ with NICS_π_(1) (R^2^ = 0.85883, with no significant improvement from second-order polynomial fit to R^2^ = 0.85909), but not with NICS_πZZ_(1) (R^2^ = 0.76060), although the latter considerably improves upon a second-order polynomial fit (R^2^ = 0.81231), as suggested by Stanger.^41^ Is important to note that the most reliable aromaticity descriptor ∫NICS_πZZ_, according to Stanger, does not correlate with any other parameter, excepting roughly with ∫NICS (R^2^ = 0.68831). The other significant correlations are those of ∫NICS with FiPC-NICS (R^2^ = 0.84501) and NICS_πZZ_(1) (R^2^ = 0.79885). The NRR appears to be the sole parameter that does not satisfactorily correlate with any other aromaticity-related descriptor and should therefore be deprecated.

In order to explore the origin of the magnetic shielding observed along the *z*-axis, an alternative NICS-related criterion was investigated. This approach involves plotting NICS-XY scans along xy planes at various z distances above the molecular plane,^44^ as well as utilizing Kleinpeter’s isochemical shielding surfaces (ICSS).^45^ The technique entails constructing isochemical shielding contour plots (ISCP) in a plane parallel to the ring plane, at a distance of 2 Å where off-center effects can be disregarded. This is achieved by employing the π-only σ_πZZ_ component, which is derived from the magnetic shielding tensor ZZ component of the aromatic molecule (σ_ZZ_) by subtracting at every point of the plane that of the perhydrogenated SOM derivative (σ_SOM_ZZ_).^40^ In the case of both reference aromatic compounds, benzene (a) and borirene (b), the highest-value ISCP lines extend over the entire ring structure (although with a slight shift toward the C=C moiety in the case of borirene). This is due to the diatropic ring currents arising from the π­(C=C) electrons which are responsible for the magnetic shielding response (Figure 8). The ISCP lines for the cyclopropenylium cation are symmetrically centered at the ring, but have a rather low σ_πZZ_ maximum (σ_πZZ_(2Å)_max_ = 0.86 ppm; compare to 14.17 and 5.83 ppm for benzene and borirene, respectively) due to the attractive effect toward the ring plane of the overall positive charge on the π electron density (see Figure S3). Thiirene displays a characteristic antiaromatic behavior, evidenced by a minimum (σ_πZZ_(2Å)_min_ = −5.92 ppm) that occurs near the ring centroid (at 0.32 Å distance). This minimum is not directly observable with the σ_πZZ_ scale employed in this study for visualization (see Figure S3). With the exception of *Al*_2_ *O*, and to a certain extent also *Ga*_2_ *O* and *Al*_2_ *S*, all other *Tr_2_C* *h* rings exhibit the central isocontour lines, which mostly involve the ring, albeit shifted toward the chalcogen atom, and show increasing maximum σ_πZZ_(2Å)_max_ values (in ppm) in the series *B*_2_ *O* (1.39) < *Ga*_2_ *O* (1.61) < *B*_2_ *S* (2.73) < *Al*_2_ *S* (2.93) < *Ga*_2_ *S* (2.97) < *B*_2_ *Se* (3.25) < *Al*_2_ *Se* (3.34) < *Ga*_2_ *Se* (3.36) < *Ga*_2_ *Te* (3.94) < *Al*_2_ *Te* (4.00) < *Ga*_2_ *Po* (4.22) < *Al*_2_ *Po* (4.29) < *B*_2_ *Te* (4.31) < *B*_2_ *Po* (4.70). This aromaticity-related parameter increases on going down the chalcogen group for every triel. This tendency is maintained within the triel group only for S and Se, but inverted for Te and Po. In the case of *Al*_2_ *O*, the ISCP exhibits the lowest-value maximum (σ_πZZ_(2Å)_max_ = 1.30 ppm) with a local maximum outside the ring projection at the z = 2Å plane, most likely arising from the highly deformed (high-energy) banana-type σ­(Al–Al) bond, constituting the HOMO, as well as the in-phase π-type overlap of the formally empty though significantly populated (0.11 e, LP*) p-type AO at both Al atoms, constituting the LUMO ().

**Figure 8:**
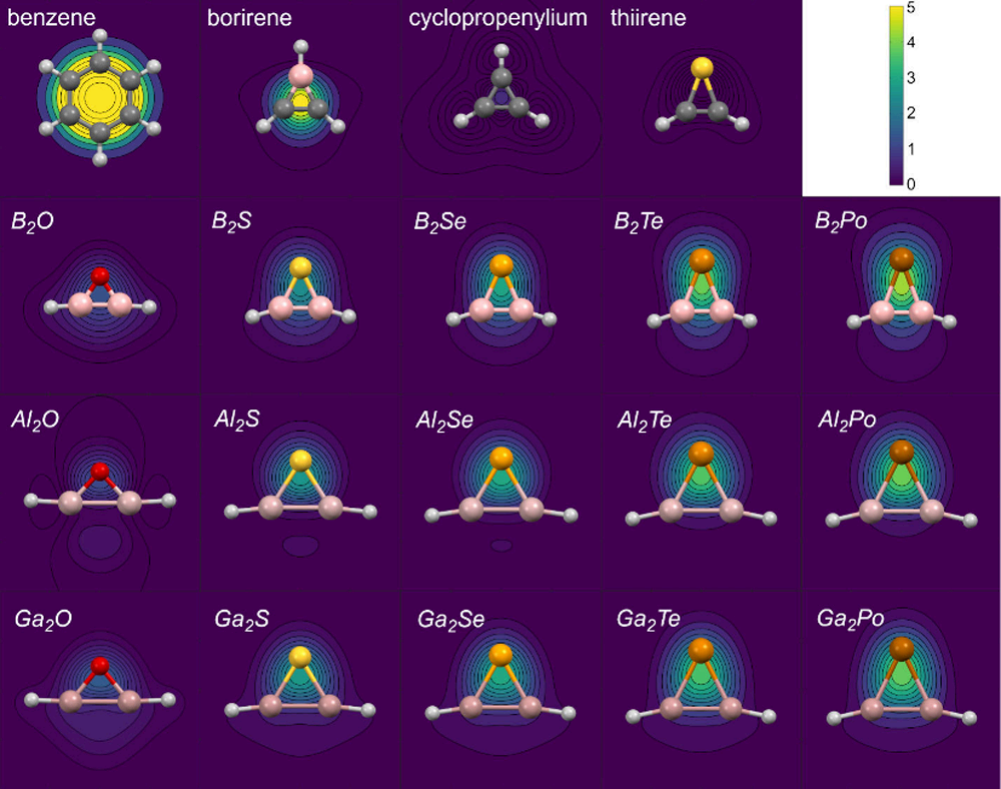
ISCP of σ_πZZ_ (ppm) in a plane parallel to the xy (ring) plane and situated 2 Å above for reference (top) and Tr_2_ *Ch* rings.

In sum, all aromaticity-related indices suggest significant aromaticity in all chalcogenaditrieliranes *Tr_2_C* *h*, except for *Al*_2_ *O*, which shows little or no aromaticity depending on the criterion used. This suggests that the RSE values of *Tr_2_C* *h* rings presented herein (Table 1) should be interpreted with caution, as they are inherently subject to contamination from resonance effects. Research is currently being conducted to explore the potential for disentangling the effects of strain and aromaticity in these (and other) rings.

### D. Updated Additive Estimation of Ring Strain Energies

Using the additivity of the strain contributions of ring atoms and/or bonds, a new efficient methodology for the rapid estimation of sufficiently reliable RSE values was recently reported.^3,6^ The 20 elements contained in the full set of p-block elements in groups 13–16, and from the second to sixth rows, could form up to 1540 different 3MRs, i.e. the combinations with repetition of 20 elements taken in groups of three. If the most metallic elements In, Th and Pb are not considered, due to their reluctance to form proper 3MRs,^2^ then the possible number of 3MRs with the 17 remaining elements decreases to 969. Making use of the already reported accurate RSE values of 137 3MRs, the additive method of atom strain contributions can be employed for the estimation of all these 969 three-membered rings, although with rather low accuracy (RMSE = 4.37 kcal/mol; MAE = 3.31 kcal/mol).^6^ The method based on a summation of single bond strain contributions can currently be applied to any saturated three-membered ring containing only 108 bonds so far. The current set of bonds include El-El, El-Tt (Tt = C, Si, Ge) and El-Pn (Pn = N, P, As, Sb) bonds, where ‘El’ is one of the 17 p-block elements excluding In, Th and Pb, and allows the significantly more accurate (RMSE = 1.085 kcal/mol; MAE = 0.62 kcal/mol) additive RSE estimation of 771 rings (80.0% of the maximum number of 969 possible 3MRs).^6^

The addition in the present study of accurate RSE values (computed from homodesmotic – RC4 – reactions) for 95 new **1** ^**El**^_**Ch**_ (*El*_2_ *Ch*) rings (all five *Ch*_3_ rings were already reported^3^), together with a set of 13 additional previously reported rings (*AlCGe*, *AlSiGe*, *GaCGe*, *GaSiGe*, *CSiO*, *CSiS*, *CGeO*, *SiGeO*,^3^ *CSiN*,^3,46^ *CNO*, *AlSiN*,^6^ *CNP*,^15b,47^ *CPO* ^6,15a^ ), make a total of two hundred forty 3MRs. Proceeding similarly to previous reports, the first approximation would use an oversized system of two hundred forty equation (eq 1) with twenty unknowns, the atom strain addends *A*, corresponding to the total number of atoms in the four groups (13–16) of the p-block.

1 $${{RSE}_{A}}^{add}=\overset{3}{\underset{i=1}{\sum }}A_{1i}$$

The set of atom-strain parameters *A*_1_^*El*^ (Figure 9, Table S2) was obtained by resolving the system of equations with a Root Mean Square Error (RMSE) of 5.37 kcal/mol (R^2^ = 0.7336). This outcome aligns with the notably scattered plot of estimated *RSE*_*A*_^*add*^ against the accurately (RC4-based) computed RSE (see Figure S5). The atom-strain contributions *A*_1_^*El*^ are consistent with the prevailing tendencies documented^2^ for heterocycles comprising a single heteroatom, and they approximately replicate the observed trends in the previously illustrated chalcogen-averaged RSE plot for *El*_2_ *Ch*, **1** ^**El**^_**Ch**_ (Figure 3). Second-row elements (particularly B, C and N) exhibit analogous values. As the groups go down, the atomic strain contributions decrease for elements with LPs (groups 15 and 16), especially for pnictogens, while they increase for those without LPs (groups 13 and 14), with the highest values observed for triels, bearing an empty p orbital (Figure 9). This effect is most likely related to the previously reported LP strain releasing effect,^2,3,6^ which in turn affects the p-character of the AOs involved in endocyclic bonds.

**Figure 9:**
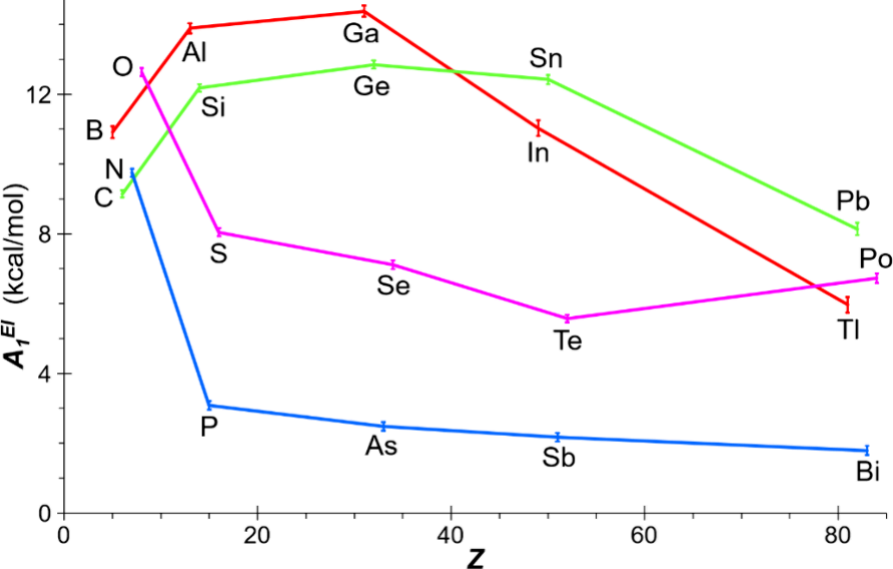
Variation of the calculated atom-strain contributions *A*_1_^*El*^ (kcal/mol) to *RSE*_*A*_^*add*^ with the atomic number. Vertical lines at each point indicate the standard deviations.

A refinement of the methodological approach involves the utilization of bond-related *B*_2_^*El*^ addends in the additive estimation *RSE*_*B*_^*add*^ (eq 2), as opposed to the employment of atom-based parameters.

2 $${{RSE}_{B}}^{ad}=\overset{3}{\underset{i=1}{\sum }}B_{2i}$$

The two hundred forty equation system, which is overdimensional due to the presence of one hundred sixty-one *B*_2_^*El‑El’*^ variables, is resolved (Table 3) resulting in a remarkably lower RMSE of 1.21 kcal/mol (R^2^ = 0.9864), which is indicative of a superior estimation resulting from this approximation (Figure 10). A limited number of the set of two hundred forty 3MRs have an additively estimated value of RSE significantly different (>2.5 kcal/mol) from the accurately (RC-4) calculated one, and almost all of them have a C–Te, C–Po, Si–N or Si–O bond (Figure 10).

**Table 3:** Bond Strain Contributions *B*_*2*_^*El‑El’*^ (kcal/mol) Obtained for the Additive RSE Estimation Methodology

	B	Al	Ga	In	Tl	C	Si	Ge	Sn	Pb	N	P	As	Sb	Bi	O	S	Se	Te	Po
B	29.38																			
Al	26.62	20.80																		
Ga		25.00	29.56																	
In				12.91																
Tl					8.58															
C	14.97	17.72	17.57			8.31														
Si		13.05	14.33			14.26	11.86													
Ge		13.61	14.92			14.13	12.59	12.81												
Sn						13.44	11.89	12.28	12.11											
Pb										5.07										
N	3.74	8.16	5.38			8.77	15.93	15.50	13.71		4.85									
P	4.01	5.45	3.46			6.16	8.50	8.46	8.81		11.50	1.68								
As	5.17	5.65	3.90			5.15	7.30	7.90	8.41		8.88	2.13	1.76							
Sb		5.61				4.45	6.30	6.95	7.52		8.21	2.49	2.22	2.24						
Bi		7.17				2.99	5.05	5.59	6.68		7.10	2.26	2.25	2.04	2.31					
O	1.01	10.43	5.88	12.68	8.19	7.75	14.99	14.18	14.43	15.60	7.97	9.64	9.20	10.14	9.43	8.20				
S	–4.29	1.75	0.20	8.55	5.52	4.69	6.41	7.98	8.14	11.61	11.46	3.69	3.80	3.86	4.02	14.50	9.93			
Se	–4.74	0.83	–0.61	7.85	5.23	4.55	5.78	6.56	7.43	11.02	9.63	3.64	3.79	3.90	3.90	13.61	9.07	8.87		
Te	–5.19	–0.55	–1.89	6.95	4.72	4.67	5.27	6.06	6.98	10.16	9.63	4.06	3.85	4.07	3.83	13.71	8.42	7.93	8.57	
Po	–6.00	–1.16	–2.57	6.30	4.19	3.79	4.71	5.41	6.43	9.67	7.73	3.91	3.65	3.61	3.72	11.25	8.25	7.49	6.91	8.25

**Figure 10:**
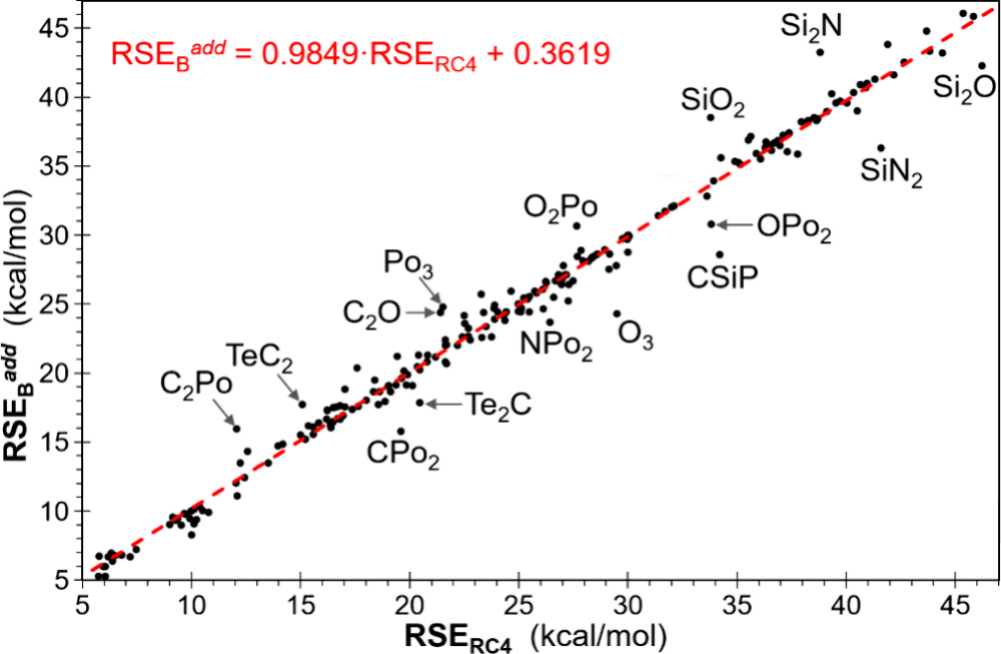
Plot of the additive *RSE*_*B*_^*add*^ versus the accurately estimated (RC-4) RSE for all 240 three-membered rings used. Rings with an absolute deviation beyond 2.5 kcal/mol are indicated.

It is important to emphasize some general trends observed in bond strain contributions. Triel-triel bonds are observed to be highly strained, particularly for the three lighter elements, consistent with the reported failure to locate the corresponding cyclic tritrieliranes *Tr*_*3*_.^3^ The same triels also form strained bonds with tetrels and bonds with negative strain contributions with chalcogens, notably the heaviest ones. A negative bond strain contribution should be understood as an extra stabilization when taking part in a 3MR and this could be an artifact related to the fact that *B*_*2*_^*Tr‑Ch*^ addends were derived from aromatic *Tr_2_C* *h* rings. Tetrel-tetrel bonds have been also found to be strained, as have those of tetrels, especially the heaviest ones, with N and O, the bond strain decreasing with other pnictogens and chalcogens as they descend in their groups. Finally, chalcogen-chalcogen bonds are also relatively strained, especially for the lightest elements.

A testbed of seven new saturated 3MRs, namely *AlCGe*, *CGeSe*, *CNS*,^48^ *CPS*,^49^ *CPSe*,^50^ *SiPS* and *SnAsTe* (Table 4), was utilized exclusively for the purpose of evaluating the performance of the additive methods, as these rings were not included in the set of 240 rings participating in the equations systems. The accurate RSE values resulting from the evaluation of RC4-type homodesmotic reactions (Scheme 1) were then compared with the *RSE*_*A*_^*add*^ estimation (Table 3). Despite the approach being rudimentary, the estimation method provides values for the RSE with a maximum unsigned absolute error (unsigned difference) of 10.42 kcal/mol. For instance, for the additive estimation of the RSE for thiaphosphirane (*CPS*), the atomic strain contributions *A*_*1*_ (in kcal/mol) for C (9.14), P (3.08) and S (8.05) are summed up, resulting in *RSE*_*A*_^*add*^ = 20.27 kcal/mol, which overestimates in 6.52 kcal/mol the recently reported^45^ reference most accurately computed RSE value of 13.75 kcal/mol, obtained by evaluation of homodesmotic reactions (Scheme 1).

**Table 4:** Calculated (DLPNO-CCSDT/def2TZVPPecp) Accurate (RC4) and Additively Estimated (add) RSEs (kcal/mol) for a Testbed of 3MRs^a^

	RSE_RC4_	RSE_A_^add^	RSE_B_^add^
**AlCGe**	46.30	35.88 (−10.42)	44.80 (−1.50)
**CGeSe**	25.21	29.11 (3.90)	25.13 (−0.08)
**CNS**	23.18	26.94 (3.76)	24.57 (1.39)
**CPS**	13.75	20.27 (6.52)	14.10 (0.35)
**CPSe**	15.66	19.34 (3.68)	14.01 (−1.65)
**SiPS**	18.58	23.31 (4.73)	18.51 (−0.08)
**SnAsTe**	18.74	20.48 (1.74)	19.24 (0.80)

a In parentheses the signed absolute errors.

For the same ring, the bond-based additive estimation would arise from summation of the bond strain contributions *B*_*2*_ (in kcal/mol) for the C–P (5.86), C–S (4.58) and P–S (3.66), resulting in *RSE*_*B*_^*add*^ = 14.10 kcal/mol, which only overestimates in 1.50 kcal/mol the reference value. Using this method, the maximum unsigned difference is only 1.65 kcal/mol for the selected set of 3MRs.

A third level for further refinement of the additive methodology would consist of using both atom- and bond-based addends in the ring strain estimation *RSE*_*A* *&B*_^*add*^ (eq 3). The resulting overdimensional system of two hundred forty equations with hundred eighty-one unknowns has a solution that only slightly outperforms that obtained with the only bonds method, as evidenced by the little improvement in the RMSE to 1.19 kcal/mol (R^2^ = 0.9870).

3 $${{RSE}_{A\&B}}^{add}=\overset{3}{\underset{i=1}{\sum }}{(A}_{3i}+B_{3i})$$

Notwithstanding the fact that it provides the most mathematically sound solution for the additive estimation of RSEs, the resulting parameters *A*_*3*_^*El*^ and *B*_*3*_^*El‑El’*^ (Table S4) appear to have lost the physical significance of the two previous approaches based on atom-only or bond-only, as well as entailing a higher level of complexity that is not justified by the minor gain in accuracy. Consequently, it is recommended to refrain from this third atom- and bond-based approach in favor of the sufficiently accurate bond-only approach. It is worth emphasizing that the above-mentioned additive methodology, based on the extended set of 161 bond-strain parameters reported here, allows the rapid and reliable estimation of the RSE for 885 rings, which represents 57.5% of the maximum number of 1540 possible 3MRs that could be formed using the 20 group 13–16 elements from the second to the sixth row. Many of the remaining 3MRs correspond to pseudorings (lacking an RCP), such as the *Tl*_*2*_ *Ch* rings mentioned in section A, or some other previously reported rings containing either two triels^3^ or the most metallic (In, Tl, Pb) elements.^2^ However, the estimated *RSE*_*B*_^*add*^ of some other rings are not yet accessible due to the existence of some white spots in the estimation of bond-strain contributions (Table 3), most of them involving bonds to In, Tl or Pb.

## Conclusions

Homodesmotic reactions and state-of-the-art computational methods were used to calculate accurate ring strain energy (RSE) values for ninety-five parent chalcogenirane rings with two other identical p-block elements, *El*_*2*_ *Ch*, excluding *Tl*_*2*_ *Ch* that were shown to form pseudorings instead. Only properly cyclic *Tl*_*2*_ *Ch* structures were found as second-order saddle points between two degenerated pseudorings. The main general electronic factor affecting the RSE was found to be the p character of the AO used in endocyclic bonds, although destabilization of the weak chalcogen-chalcogen bonds plays an important role for the subset of *Ch*_*2*_ *Ch’* rings studied here. The expected 2π-electron Hückel aromaticity in chalcogenaditrieliranes *Tr_2_C* *h* is a second important electronic factor influencing stability and thus ring strain. Some qualitative and quantitative criteria based on NICS were used to establish an order of aromaticity within the *Tr_2_C* *h* rings, which was generally found to be 1) increasing with decreasing chalcogen group, 2) lower for Al compared to B and Ga, and 3) slightly varying in the order B > Ga for the heaviest chalcogens but vice versa for the lightest ones. According to most of these criteria, *Al*_*2*_ *O* and *B*_*2*_ *Po* are the least and most aromatic, respectively, among the *Tr_2_C* *h* rings.

The additive method, based on the extended set of one hundred and sixty-one bond strain parameters reported here, allows the rapid estimation of the RSE for 885 rings (57.5% of the maximum number of 3MRs that could be formed from all twenty Group 13–16 elements from the second to the sixth row) with remarkable accuracy (RMSE = 1.21 kcal/mol; MAE = 0.69 kcal/mol). The more complex and less physically meaningful combined atoms-and-bonds method only slightly outperforms the bond only method, so its use is not recommended.

## Experimental Section

Density functional theory (DFT) calculations were performed with the ORCA program.^51^ All geometry optimizations were run in redundant internal coordinates in the gas phase, with tight convergence criteria, and using the B3LYP^52^ functional together with Ahlrichs segmented def2-TZVP basis set^53^ and the latest Grimme’s semiempirical atom-pairwise London dispersion correction (DFT-D4).^54^ From these geometries, all electronic data were obtained through single-point calculations (SP) using a higher quality basis set including additional polarization, def2-QZVPP.^55^ Relativistic effects for elements in the fifth and sixth rows were considered by employing the def2-ECP effective core potentials as included in Orca by default. Energy values were corrected for the zero-point vibrational term at the optimization level and obtained by the newly developed DLPNO method^56^ for the “coupled-cluster” level with single, double, and triple perturbatively introduced excitations (CCSD­(T)).^57^ Analysis of the hybridization in the AO used for the endocyclic bonds was performed with the NBO method.^58^ Properties derived from the topological analysis of the electronic density were obtained with the Multiwfn program.^59^ Grid points for calculation of NICS-related properties, isochemical shielding contour plots and the mathematical solution of the systems of equations for the additive methodology were done with GNU Octave scripts.^60^ NICS variation plots along the axis orthogonal to the ring plane (z) where computed from 0 to 5 Å, in Δr = 0.002 Å intervals, starting at the ring centroid. Isochemical shielding contour plots were computed in a plane parallel to the ring (xy) plane, at 2 Å distance, taking points from −4.00 to +4.00 Å in 0.08 Å intervals in both x and y directions (a grid of 10200 equi-spaced points).

## Supplementary Material





## Notes

Additional figures and tables. Cartesian coordinates and energies for all computed species (PDF)
Cartesian coordinates for all computed species (XYZ)

## Abbreviations

3MRs: three-membered rings
RSE: ring strain energy
RC4: reaction class 4 (homodesmotic)
RCP: ring critical point
BCP: bond critical point
NBO: natural bond orbital
SOPT: second-order perturbation theory
WBI: Wiberg bond index
VSCC: valence-shell charge concentration
NICS: nucleus-independent chemical shift
NRR: NICS rate ratio
FiPC-NICS: free of in-plane component NICS
ISCP: isochemical shielding contour plot

## References

[1] He G., Shynkaruk O., Lui M. W., Rivard E. (2014). Small Inorganic Rings
in the 21st Century: From Fleeting Intermediates to Novel Isolable
Entities. Chem. Rev..

[2] Planells A. R., Ferao A. E. (2020). Accurate ring strain
energy calculations on saturated
three-membered heterocycles with one group 13–16 element. Inorg. Chem..

[3] Rey
Planells A., Espinosa Ferao A. (2022). Ring Strain Energies of Three-membered
Homoatomic Inorganic rings El_3_ and Diheterotetreliranes
El_2_Tt (Tt = C, Si, Ge). Accurate versus Additive Approaches. Inorg. Chem..

[4] b Rebsdat, S. ; Mayer, D. Ullmann’s encyclopedia of industrial chemistry, 7th ed.; Wiley-VCH: Weinheim, Wiley online library, 2010.

[5] Pham, H. Q. ; Marks, M. J. Ullmann’s encyclopedia of industrial chemistry, 7th ed.; Wiley-VCH: Weinheim, Wiley online library, 2010.

[6] Espinosa
Ferao A., Rey Planells A. (2023). Ring Strain Energy of Diheteropnictogeniranes
El_2_Pn (Pn = N, P, As, Sb) – Accurate versus Additive
Approaches. Chem.—Eur. J..

[7] Saito M., Tokitoh N., Okazaki R. (2024). Main Group
Analogs of Dichalcogeniranes. Eur. J. Inorg.
Chem..

[8] Sander W., Schroeder K., Muthusamy S., Kirschfeld A., Kappert W., Boese R., Kraka E., Sosa C., Cremer D. (1997). Dimesityldioxirane. J. Am. Chem. Soc..

[9] Schreiner P. R., Reisenauer H. P., Romanski J., Mloston G. (2010). Oxathiirane. J. Am. Chem. Soc..

[10] Sawwan N., Greer A. (2007). Rather Exotic Types
of Cyclic Peroxides: Heteroatom Dioxiranes. Chem. Rev..

[11] Ho D. G., Gao R., Celaje J., Chung H.-Y., Selke M. (2003). Phosphadioxirane: A
Peroxide from an Ortho-Substituted Arylphosphine and Singlet Dioxygen. Science.

[12] Tokitoh N., Sadahiro T., Hatano K., Sasaki T., Takeda N., Okazaki R. (2002). Synthesis of Kinetically Stabilized Silaneselone and
Silanetellone. Chem. Lett..

[13] Saito M., Tokitoh N., Okazaki R. (1997). The First
Kinetically Stabilized
Stannaneselone and Diselenastannirane: Synthesis by Deselenation of
a Tetraselenastannolane and Structures. J. Am.
Chem. Soc..

[14] Mardyukov A., Keul F., Schreiner P. R. (2019). Preparation and Characterization
of Phenyl Phosphine Diselenide – The Monomeric Form of Woollins’
Reagent. Eur. J. Org. Chem..

[15] Espinosa Ferao A., Rey Planells A., Streubel R. (2021). Between oxirane and phosphirane: the spring loaded
oxaphosphirane ring. Eur. J. Inorg. Chem..

[16] Rey
Planells A., Espinosa Ferao A. (2022). Accurate Ring Strain Energies of
Unsaturated Three-Membered Heterocycles with One Group 13–16
Element. Inorg. Chem..

[17] Stephan D. W., Erker G. (2015). Frustrated Lewis Pair
Chemistry: Development and Perspectives. Angew
Chem Int Ed.

[18] Villalba Franco J. M., Schnakenburg G., Sasamori T., Espinosa
Ferao A., Streubel R. (2015). Stimuli-Responsive
Frustrated Lewis-Pair-Type Reactivity of a Tungsten Iminoazaphosphiridine
Complex. Chem.—Eur. J..

[19] Espinosa
Ferao A. (2024). Carbonylation of Boranes – A Computational Study. Eur. J. Inorg. Chem..

[20] Wheeler S. E., Houk K. N., Schleyer P. v. R., Allen W. D. (2009). A Hierarchy of Homodesmotic
Reactions for Thermochemistry. J. Am. Chem.
Soc..

[21] a Bader, R. F. W. Atoms in Molecules: A Quantum Theory; Oxford University Press, Oxford, 1990.

[22] Glendening, E. D. ; Badenhoop, J. K. ; Reed, A. E. ; Carpenter, J. E. ; Bohmann, J. A. ; Morales, C. M. ; Weinhold, F. Theoretical Chemistry Institute; University of Wisconsin, Madison, 2001.

[23] Stanford M. W., Schweizer J. I., Menche M., Nichol G. S., Holthausen M. C., Cowley M. J. (2019). Intercepting the Disilene-Silylsilylene Equilibrium. Angew. Chem., Int. Ed..

[24] Cotton, F. A. ; Wilkinson, G. ; Murillo, C. A. ; Bochmann, M. Advanced Inorganic Chemistry, 6th ed.; Wiley-Interscience: 1999.

[25] a Luo, Y.-R. Comprehensive Handbook of Chemical Bond Energies; Imprint CRC Press: Boca Raton, FL, 2007.

[26] Greenwood, N. N. ; Earnshaw, A. Chemistry of the Elements, 2nd ed.; Butterworth-Heinemann: 1997.

[27] Yang Y.-M., Yu H.-Z., Sun X.-H., Dang Z.-M. (2016). Density functional
theory calculations on S―S bond dissociation energies of disulfides. J. Phys. Org. Chem..

[28] Steudel, R. Chemistry of the Non-Metals: Syntheses - Structures - Bonding – Applications; De Gruyter: Berlin, Boston, 2020.

[29] Irigoyen M., Fernández A., Ruiz A., Ruipérez F., Matxain J. M. (2019). Diselenide Bonds
as an Alternative to Outperform the
Efficiency of Disulfides in Self-Healing Materials. J. Org. Chem..

[30] Mugesh G., du Mont W.-W., Sies H. (2001). Chemistry of Biologically Important
Synthetic Organoselenium Compounds. Chem. Rev..

[31] Irgolic, K. J. Tellurium. In Comprehensive Coordination Chemistry; Wilkinson, G. , Ed.; Pergamon Press: Oxford, 1992.

[32] Liu Y., Li Y., Yang Q., Yang J.-D., Zhang L., Luo S. (2024). Prediction
of Bond Dissociation Energy for Organic Molecules Based on a Machine-Learning
Approach. Chin. J. Chem..

[33] Paetzold P., Géret-Baumgarten L., Boese R. (1992). Bis­(trisyl)­oxadiborinane. Angew. Chem., Int.
Ed. Engl..

[34] Zhu Q., Chen S., Chen D., Lin L., Xiao K., Zhao L., Solà M., Zhu J. (2023). The application of
aromaticity and antiaromaticity to reaction mechanisms. Fundam. Res..

[35] Hofmann M., von Rague Schleyer P., Regitz M. (1999). The Structures and
Energies of Phosphaalkyne
Trimers, (HCP)_3_. Eur. J. Org. Chem..

[36] Schleyer P. v. R., Jiao H., Hommes N. J. R. v. E., Malkin V. G., Malkina O. L. (1997). Nucleus-Independent
Chemical Shifts: A Simple and Efficient Aromaticity Probe. J. Am. Chem. Soc..

[37] Janda T., Foroutan-Nejad C. (2018). Why is Benzene
Unique? Screening Magnetic Properties of C_6_H_6_ Isomers. ChemPhysChem.

[38] Stanger A. (2006). Nucleus-Independent
Chemical Shifts (NICS): Distance Dependence and Revised Criteria for
Aromaticity and Antiaromaticity. J. Org. Chem..

[39] Noorizadeh S., Dardab M. (2010). A new NICS-based aromaticity
index; NICS-rate. Chem. Phys. Lett..

[40] Torres-Vega J. J., Vásquez-Espinal A., Caballero J., Valenzuela M. L., Alvarez-Thon L., Osorio E., Tiznado W. (2014). Minimizing
the Risk of Reporting False Aromaticity and Antiaromaticity in Inorganic
Heterocycles Following Magnetic Criteria. Inorg.
Chem..

[41] Stanger A. (2019). Reexamination
of NICSπ,zz: Height Dependence, Off-Center Values, and Integration. J. Phys. Chem. A.

[42] Cordero B., Gómez V., Platero-Prats A. E., Revés M., Echeverría J., Cremades E., Barragán F., Alvarez S. (2008). Covalent radii revisited. Dalton
Trans..

[43] a Housecroft, C. E. ; Sharpe, A. G. Inorganic Chemistry, 3rd ed.; Pearson Education: 2008.

[44] Gershoni-Poranne R., Stanger A. (2014). The NICS-XY-Scan: Identification of Local and Global
Ring Currents in Multi-Ring Systems. Chem.—Eur.
J..

[45] Klod S., Kleinpeter E. (2001). *Ab
initio* calculation of the anisotropy effect of multiple bonds
and the ring current effect of arenes—application in conformational
and configurational analysis. J. Chem. Soc.,
Perkin Trans..

[46] Chen W., Wang L., Li Z., Lin A., Lai G., Xiao X., Deng Y., Kira M. (2013). Diverse reactivity
of an isolable dialkylsilylene toward imines. Dalton Trans..

[47] Espinosa A., Streubel R. (2011). Computational studies on azaphosphiridines and the
quest of how to effect ring-opening processes via selective bond activation. Chem.—Eur. J..

[48] Schaumann E., Bolte O., Behr H. (1990). S-Vinyl thio-oximes from thioketenes
and benzylnitrene. J. Chem. Soc. Perkin Trans.
1.

[49] Espinosa
Ferao A., Streubel R. (2016). Thiaphosphiranes and their complexes:
systematic study on ring strain and ring cleavage reactions. Inorg. Chem..

[50] Sase S., Kano N., Kawashima T. (2002). Synthesis
and Structure of the First
1,2σ5-Selenaphosphirane. J. Am. Chem.
Soc..

[51] Neese, F. ORCA - An Ab initio, DFT and Semiempirical SCF-MO Package, version 4.2.1; Max Planck Institute for Bioinorganic Chemistry, 2019.

[52] Lee C., Yang W., Parr R. G. (1988). Development
of the Colle-Salvetti correlation energy formula into a functional
of the electron density. Phys. Rev. B.

[53] Weigend F., Ahlrichs R. (2005). Balanced basis sets
of split valence, triple zeta valence
and quadruple zeta valence quality for H to Rn: Design and assessment
of accuracy. Phys. Chem. Chem. Phys..

[54] Caldeweyher E., Mewes J.-M., Ehlert S., Grimme S. (2020). Extension and evaluation
of the D4 London-dispersion model for periodic systems. Phys. Chem. Chem. Phys..

[55] Schäfer A., Huber C., Ahlrichs R. J. (1994). Fully optimized
contracted Gaussian basis sets of triple zeta valence quality for
atoms Li to Kr. Chem. Phys..

[56] Riplinger C., Sandhoefer B., Hansen A., Neese F. (2013). Natural triple excitations
in local coupled cluster calculations with pair natural orbitals. J. Chem. Phys..

[57] Pople J. A., Head-Gordon M., Raghavachari K. (1987). Quadratic configuration interaction.
A general technique for determining electron correlation energies. J. Chem. Phys..

[58] Reed A. E., Weinhold F. (1983). Natural bond orbital
analysis of near-Hartree–Fock water dimer. J. Chem. Phys..

[59] Lu T., Chen F. (2012). Multiwfn: A Multifunctional Wavefunction Analyzer. J. Comput. Chem..

[60] Eaton, J. W. ; Bateman, D. ; Hauberg, S. ; Wehbring, R. ; GNU Octave version 9.4.0 manual: a high-level interactive language for numerical computations; 2023. https://www.gnu.org/software/octave/doc/v8.2.0/.

