# Supplementary material for: Unravelling the Strain: Accurate Ring Strain Energies in Chalcogeniranes and Predictive Models for Most p‑Block Three-Membered Rings
Source: Inorg Chem. 2025 Jun 3;64(23):11832–44. doi: 10.1021/acs.inorgchem.5c01736 (PMC12175124; doi:10.1021/acs.inorgchem.5c01736)
Supplement: Supplementary file 1 [file ic5c01736_si_001.pdf]

Supporting Information

Unravelling the Strain: Accurate Ring Strain Energies in Chalcogeniranes and Predictive Models for most p-Block Three-Membered Rings

Arturo Espinosa Ferao [a]

[a] Depto. Química Orgánica, Facultad de Química, Campus de Espinardo, Universidad de Murcia, 30100 Murcia (Spain), E-mail: artuesp@um.es.

Table of contents

.... Page

Figure S1. Histogram for the variability of individual RSE_i values S2

Figure S2. $\sigma_{\pi ZZ}$ variation plot for selected rings S2

Table S1. Regression coefficients for the linear correlations among all pairs of aromaticity descriptors collected in Table 2 .... S3

Figure S3. ISCP of $\sigma_{\pi ZZ}$ in a z = 2 Å plane for cyclopropenylium cation and thiirene.......................... S3

Figure S4. Computed Kohn-Sham isosurfaces for HOMO and LUMO of Al_2O. S4

Figure S5. Plot of the additive RSE_A^{add} versus the accurately estimated RSE for all 3MRs S4

Table S2. Atom strain contributions A_1^{El} obtained for the RSE_B^{add} estimation S5

Table S3. Atom (A_3^{El}) and bond ($B_3^{El\text{-}El'}$) strain contributions obtained for the $RSE_{A\&B}^{add}$ estimation... S6

Calculated structures. Cartesian coordinates and energies for all computed minima....................... S7

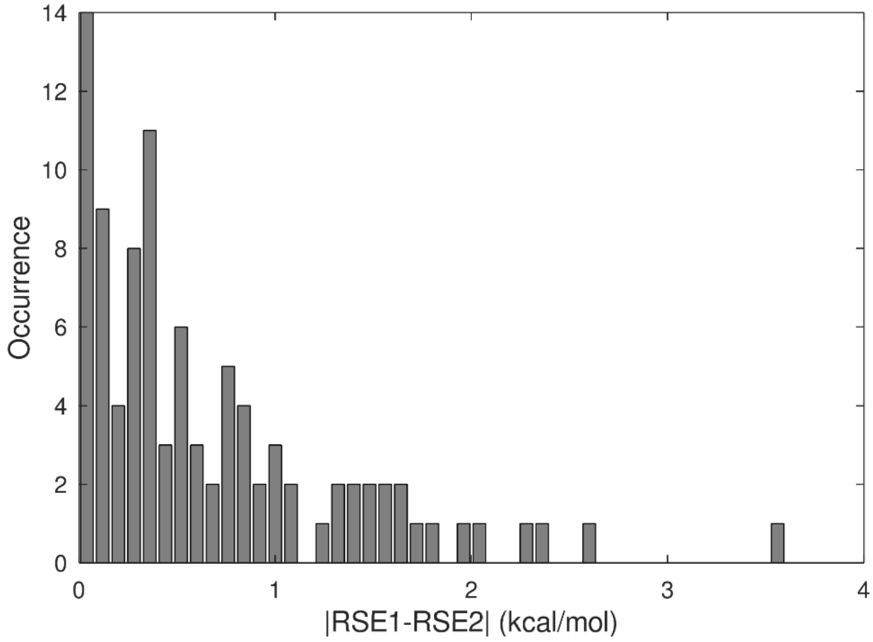

Figure S1. Histogram for the variability of the two individual energy values employed to obtain the average RSE.

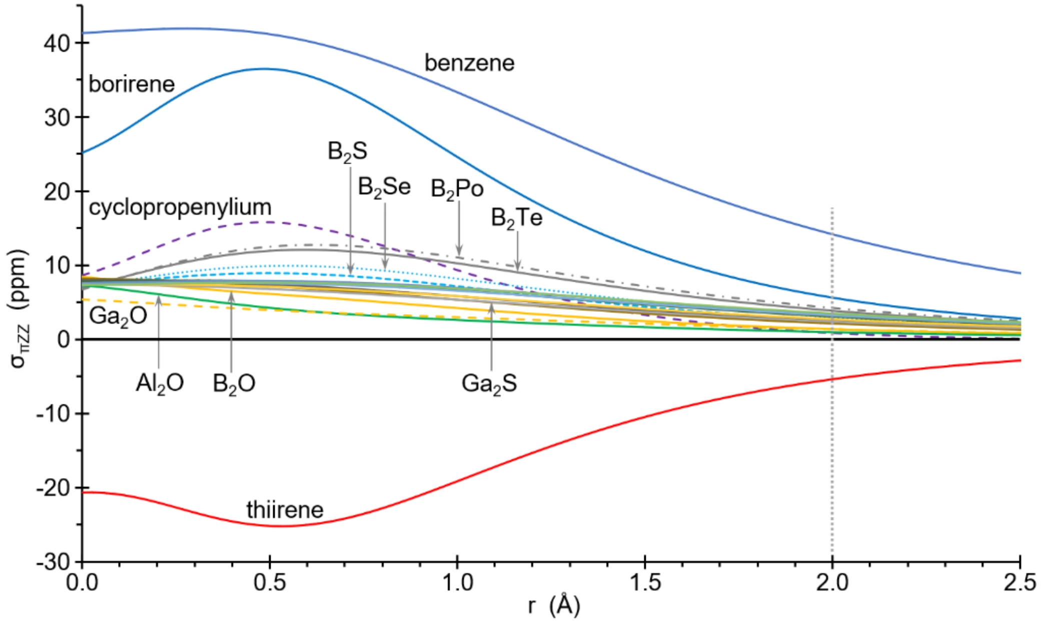

Figure S2. $\sigma_{\pi ZZ}$ variation plot along the perpendicular axis at the ring centroid for selected rings.

Table S1. Regression coefficients (R^2) for the linear correlations among all pairs of aromaticity descriptors collected in Table 2.

	NICS(1)	$NICS_{\pi}(1)$	$NICS_{ZZ}(1)$	$NICS_{\pi ZZ}(1)$	NRR	FiPC-NICS	∫NICS	$∫NICS_{\pi ZZ}$
$NICS_{ZZ}(0)$	**0.82137**	0.03716	**0.87140**	0.24499	0.19799	0.30585	0.09361	0.00179
NICS(1)		0.00180	**0.93398**	0.54184	0.34630	0.68954	0.42101	0.11153
$NICS_{\pi}(1)$			0.03427	0.47411	0.10943	0.06286	0.35167	**0.85883**
$NICS_{ZZ}(1)$				0.32158	0.32594	0.60762	0.26966	0.01463
$NICS_{\pi ZZ}(1)$					0.38180	0.62964	**0.79885**	0.76060
NRR						0.57356	0.53381	0.26685
FiPC-NICS							**0.84501**	0.31645
∫NICS								0.68831

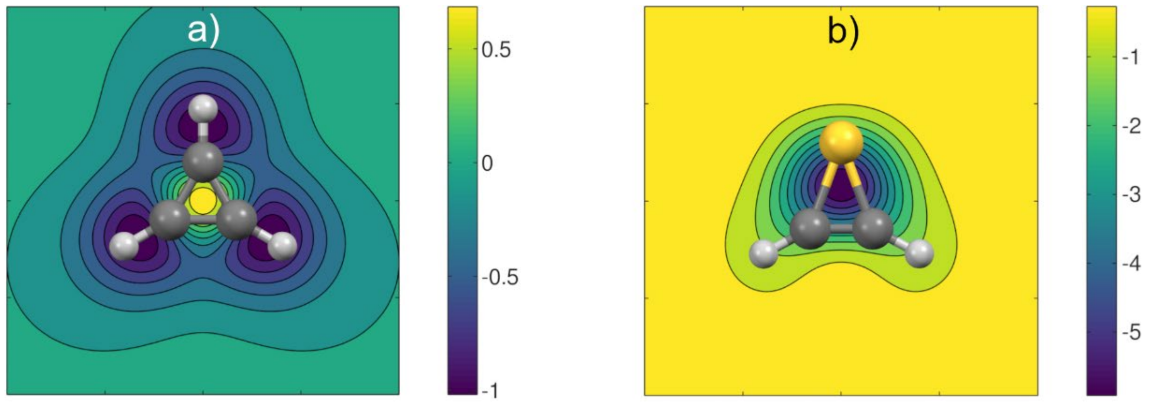

Figure S3. ISCP of $\sigma_{\pi ZZ}$ (ppm) in a plane parallel to the xy (ring) plane and situated ±2 Å above for reference a) cyclopropenylium cation and b) thiirene ring.

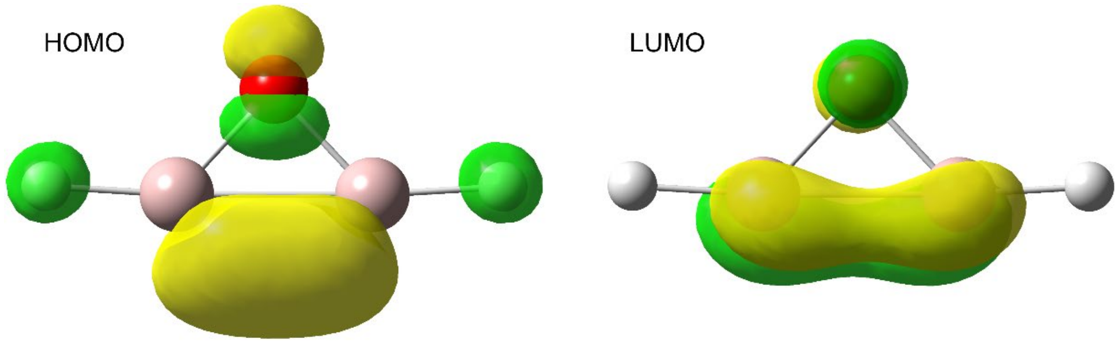

Figure S4. Computed [B3LYP/def2-TZVP] Kohn-Sham isosurfaces (0.06 au) for HOMO and LUMO of *Al_2O*.

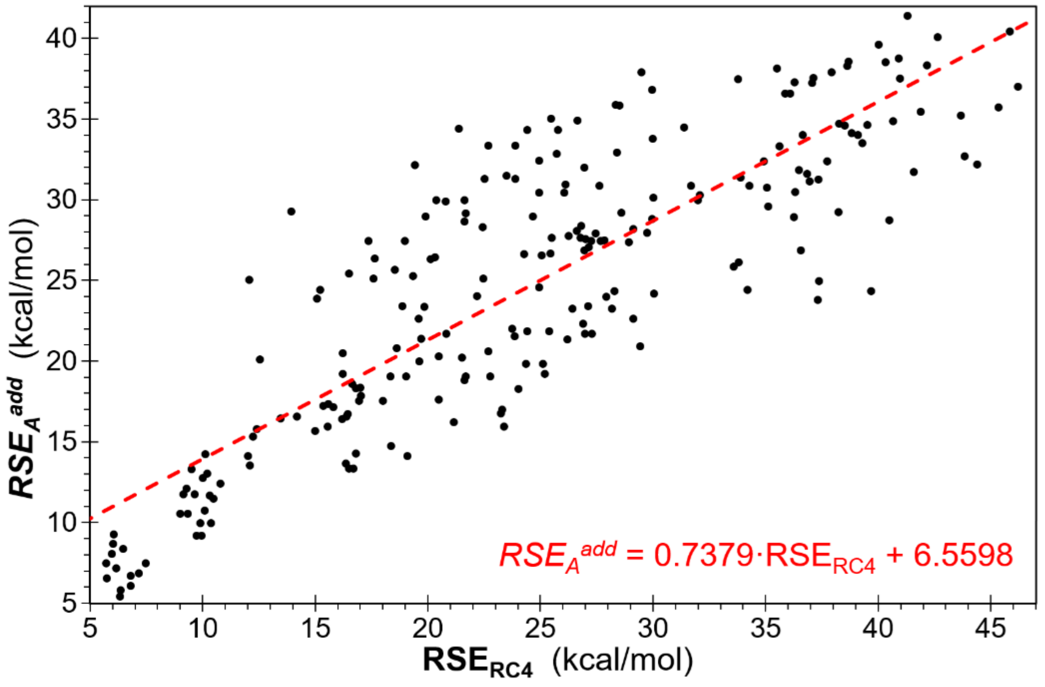

Figure S5. Plot of the additive RSE_A^{add} versus the accurately estimated (RC-4) RSE for all 240 three-membered rings used.

Table S2. Atom strain contributions A_1^{El} (kcal/mol) obtained for the additive methodology of RSE estimation.

Group 13		Group 14		Group 15		Group 16	
B	10.92	C	9.14	N	9.75	O	12.63
Al	13.89	Si	12.18	P	3.08	S	8.05
Ga	14.37	Ge	12.85	As	2.48	Se	7.11
In	11.03	Sn	12.42	Sb	2.18	Te	5.57
Tl	5.97	Pb	8.14	Bi	1.79	Po	6.73

Table S3. Atom A_3^{El} and bond strain contributions $B_3^{El\text{-}El'}$ (kcal/mol) obtained for the additive RSE estimation methodology.

	A_3^{El}	$B_3^{El\text{-}El'}$																			
		B	Al	Ga	In	Tl	C	Si	Ge	Sn	Pb	N	P	As	Sb	Bi	O	S	Se	Te	Po
B	1.55	21.27																			
Al	2.61	18.31	12.30																		
Ga	2.33		16.63	21.32																	
In	2.40				8.74																
Tl	0.93					6.04															
C	1.12	13.60	15.54	15.73			7.26														
Si	2.11		10.86	12.19			12.56	9.77													
Ge	2.12		10.96	12.58			12.40	10.57	10.83												
Sn	2.57						11.57	10.01	10.43	9.54											
Pb	1.46										3.62										
N	1.34	5.58	9.14	6.51			7.32	13.95	13.71	11.75		3.62									
P	-0.57	6.80	7.38	5.54			5.58	7.73	7.61	7.81		10.90	2.38								
As	-0.35	7.85	7.47	5.87			4.72	6.39	6.96	7.30		8.34	2.61	2.17							
Sb	0.66		6.93				3.52	4.89	5.50	5.90		7.17	2.48	2.29	1.63						
Bi	-0.26		8.94				2.53	4.12	4.66	5.52		6.56	2.67	2.55	1.83	2.57					
O	2.21	2.41	10.91	6.57	11.26	7.44	6.46	13.03	12.19	12.04	13.77	6.38	8.68	8.26	8.70	8.45	5.94				
S	0.85	-2.21	2.91	1.56	7.82	5.44	3.58	4.81	6.43	6.43	10.46	10.32	3.49	3.51	3.06	3.72	12.96	9.16			
Se	0.03	-2.24	2.40	1.17	7.52	5.56	3.94	4.69	5.31	6.12	10.28	8.91	3.87	3.91	3.53	4.01	12.49	8.60	8.88		
Te	0.29	-2.83	0.88	-0.24	6.49	4.92	3.98	4.09	4.26	5.55	9.28	8.82	4.20	3.90	3.61	3.81	12.50	7.86	7.79	8.14	
Po	2.07	-3.64	0.28	-0.92	5.84	4.39	2.29	3.17	3.83	4.99	8.80	6.56	3.69	3.33	2.79	3.70	9.69	7.33	6.99	5.44	5.71

Calculated structures

Cartesian coordinates (in Å), G correction (G-E) and ZPE (in hartrees) were computed at RIJCOSX-B3LYP-D4/def2-TZVP(ecp). For TSs the imaginary frequency is given. In addition, electronic energies (in hartrees) are quoted using the default DLPNO-CCSD(T)/def2-QZVPP(ecp) level.

B_2O E = -125.9370571052 au
ZPE = 0.02769922 au
G_{corr} = 0.00401187 au

B	1.42691527443020	0.04904701900097	-0.86228414125189
H	2.15417531867490	-0.87770985108019	-0.93715208701157
B	0.52185831560783	1.32902899235802	-0.86221891422352
H	-0.10946248086097	2.32361816416463	-0.93695274817755
O	0.39993273159316	0.28284533190073	0.03589721035373

B_2S E = -448.5319651434 au
ZPE = 0.02532387 au
G_{corr} = 0.00032074 au

B	2.56883428517002	2.25875206000819	-0.14757210383184
H	3.27381617154114	3.20237785163760	-0.13690166592785
B	2.03026102428338	0.76547655197148	-0.19006675872792
H	1.97115348302716	-0.40993501888111	-0.23971980031216
S	0.78228474541345	2.05674666084176	-0.07704838011099

B_2Se E = -2451.2203043159 au
ZPE = 0.02454587 au
G_{corr} = -0.00176886 au

B	2.62432067736528	2.23536362821364	-0.14676526004177
H	3.34805657980495	3.16398261699032	-0.13577169635599
B	2.08337491419391	0.74538963490775	-0.18788668414121
H	2.04311194303034	-0.43036223155969	-0.23494047347674
Se	0.68014376543611	2.09555455177896	-0.07926977314978

B_2Te E = -318.3461202218 au
ZPE = 0.02381895 au
G_{corr} = -0.00340143 au

B	2.65799869018953	2.21983644417708	-0.14866980692454
H	3.40250429146106	3.13140299364915	-0.13918354390272
B	2.11911060642646	0.73571253316433	-0.18958133927951
H	2.10598512131867	-0.44023539744460	-0.23775198499311
Te	0.49340917043487	2.16321162678502	-0.06944721206561

B_2Po E = -287.9978251378 au
ZPE = 0.02350295 au
G_{corr} = -0.00452246 au

B	2.68208788065401	2.20750829144173	-0.15005087357340
H	3.41703886685922	3.12614944371197	-0.13998544967178
B	2.14547926277865	0.72985686227014	-0.19074402405049
H	2.12043630358446	-0.44541317140236	-0.23856318335335
Po	0.41396556595425	2.19182677430949	-0.06529035651647

Al_2O E = -560.4075578117 au
ZPE = 0.01715099 au
G_{corr} = -0.00946779 au

Al	1.63076252755735	3.07413658501244	0.04569077309306
H	0.77596905504077	4.40614253083531	0.02848421495093
Al	2.78906429296654	1.00835358848146	0.03306052960442
H	3.48043122006392	-0.41497540819111	-0.00127430595525
O	1.14080488320951	1.44378350580176	-0.29490298007392

Al_2S E = -883.0143524927 au
ZPE = 0.01579009 au
G_{corr} = -0.01216588 au

Al	2.84842427795143	2.58392429413640	-0.14343162150299
H	3.54397781920931	3.99821715898802	-0.11246074475227
Al	2.02977712870268	0.33469887697132	-0.20497520946824
H	1.65339611393255	-1.19526311025896	-0.25441268271035
S	0.67851491044578	2.09742119958588	-0.07768555891714

Al_2Se E = -2885.7106142733 au
ZPE = 0.01523933 au
G_{corr} = -0.01399614 au

Al	2.88696821541939	2.57631752151768	-0.14248686000288
H	3.64314699092549	3.95903717098203	-0.11231810863653
Al	2.06696184939981	0.31577812109156	-0.20484095300914
H	1.75837605646130	-1.22901679687194	-0.25502733205602
Se	0.56315883097613	2.13771389972542	-0.08918118760815

Al_2Te E = -752.8428212250 au
ZPE = 0.01484777 au
G_{corr} = -0.01532078 au

Al	2.89995272503820	2.57969900095130	-0.14305562077240
H	3.73824974336509	3.91490997748021	-0.11664414558544
Al	2.07452082657188	0.30478528831147	-0.20570855717056
H	1.86008820029056	-1.25642542495336	-0.25894946352401

Te	0.34580044791639	2.21686107465512	-0.07949665426031

Al_2Po E = -722.4977666389 au
ZPE = 0.01463023 au
G_{corr} = -0.01634693 au

Al	2.90583353703545	2.57956178046676	-0.14379570365173
H	3.77498640379545	3.89567084625636	-0.11801599103731
Al	2.07910197372785	0.30107341974783	-0.20650089164834
H	1.90073470472681	-1.26526175131413	-0.26005937659172
Po	0.25795532389657	2.24878562128791	-0.07548247838362

Ga_2O E = -3923.8224659001 au
ZPE = 0.01608995 au
G_{corr} = -0.01290207 au

Ga	2.70904124282162	2.60827473414958	-0.13178884947459
H	3.18079097321645	4.09846729862833	-0.09244875305289
Ga	1.90053808686338	0.40437547466806	-0.19214209828793
H	1.30053793285853	-1.03867190810988	-0.23039352436885
O	0.96272390230826	1.99626043134890	-0.10966458307630

Ga_2S E = -4246.4635830714 au
ZPE = 0.01500965 au
G_{corr} = -0.01526313 au

Ga	2.85992213255896	2.55609079453734	-0.14324056236408
H	3.55215770773430	3.95077310539182	-0.11598690632229
Ga	2.05473744111247	0.34906913067938	-0.20400047253198
H	1.69220737603044	-1.16456015814407	-0.25389788112802
S	0.63008503692070	2.11559170721651	-0.07187372942481

Ga_2Se E = -6249.1642916145 au
ZPE = 0.01445844 au
G_{corr} = -0.01702369 au

Ga	2.91708628498339	2.55217836431903	-0.14442767711092
H	3.68064073101300	3.90920896276768	-0.11320525581075
Ga	2.08199909609130	0.35163153428376	-0.20250727033707
H	1.76253215546078	-1.17216760812460	-0.24475501255830
Se	0.53816019028567	2.19319425851671	-0.12729342044777

Ga_2Te E = -4116.3028854533 au
ZPE = 0.01406282 au
G_{corr} = -0.01830189 au

Ga	2.92767977480953	2.55180323490074	-0.14452477760206
H	3.76712426370291	3.86461846228451	-0.11542096368139

Ga	2.08974912589163	0.34537844074364	-0.20377590339404
H	1.85643028788821	-1.19470078812419	-0.24594516675379
Te	0.33943500554186	2.26694616195788	-0.12252182483353

Ga₂Po E = -4085.9598573681 au
ZPE = 0.01383048 au
G_{corr} = -0.01929464 au

Ga	2.93438049397828	2.54899479826082	-0.14495962619405
H	3.80015846480366	3.84606763352197	-0.11540026251441
Ga	2.09434640198653	0.34284437131977	-0.20752523269684
H	1.89521087338527	-1.20330544796084	-0.24423074454243
Po	0.25632222368039	2.29944415662086	-0.12007277031709

In_2O E = -455.6409535346 au
ZPE = 0.01373296 au
G_{corr} = -0.01744609 au

In	2.80507875270466	2.75538059923876	-0.13047319701955
H	3.29466013541629	4.40768063668867	-0.08315679227633
In	1.88107355406227	0.22954179760299	-0.19853524548198
H	1.18687749563194	-1.34803272650073	-0.23723355316181
O	0.88594220025307	2.02413572365528	-0.10703902032089

In_2S E = -778.2976560260 au
ZPE = 0.01290543 au
G_{corr} = -0.01942600 au

In	2.96230121548177	2.69484235886812	-0.14283108475802
H	3.66122564790721	4.26224978425582	-0.10640732624432
In	2.04307031567245	0.17740522852526	-0.21125912446360
H	1.57605667168334	-1.47341561955882	-0.26141067434736
S	0.54645584361209	2.14588282759058	-0.06709134195788

In_2Se E = -2781.0024809431 au
ZPE = 0.01241183 au
G_{corr} = -0.02118046 au

In	3.01897709869774	2.69046228065324	-0.14195892777919
H	3.79574831148023	4.22111799449131	-0.10485205754074
In	2.06830683770116	0.18113081311639	-0.20701959758529
H	1.64104914878166	-1.48050618468416	-0.25293182615950
Se	0.45633706117334	2.22184060818580	-0.12542622720008

In_2Te E = -648.1462569393 au
ZPE = 0.01205998 au
G_{corr} = -0.02123793 au

In	3.02444237982342	2.69111038521333	-0.14228158979777
H	3.88990633563032	4.17465305041847	-0.10738472268611
In	2.07069787523816	0.17686445256533	-0.20657766041534
H	1.74312186934020	-1.50847775302789	-0.25537215628564
Te	0.25224999780204	2.29989537659334	-0.12057250707994

In₂Po E = -617.8053941079 au
ZPE = 0.01187593 au
G_{corr} = -0.02212837 au

In	3.03027021686289	2.68884572120450	-0.14256122480938
H	3.92361160681065	4.15733224318365	-0.10830362790626
In	2.07147397811208	0.17518030586762	-0.20653625549282
H	1.78291865924624	-1.51857236067820	-0.25622864410181
Po	0.17214399680225	2.33125960218502	-0.11855888395455

Tl_2O* E = -420.1476983092 au
ZPE = 0.01385154 au
G_{corr} = -0.01917199 au

Tl	2.31933942441273	3.13896155880908	-0.10417900252654
H	3.58371414825330	4.21349011869112	-0.09177397632689
Tl	2.34542956821271	0.00018549946676	-0.21324213063407
H	1.03521617491765	-1.29850448480241	-0.23166276101525
O	0.76993282227182	2.01457333852040	-0.11557993775780

$[Tl_2O]^{\ddagger}$ E = -420.1363666692 au
ZPE = 0.01292445 au
G_{corr} = -0.01922798 au
$\bar{\nu}$ = -288.23 cm^{-1}

Tl	2.81581921856161	2.78627602378024	-0.12536758418133
H	3.35422046146506	4.43037168668123	-0.07837697092182
Tl	1.87029097138088	0.19922468580813	-0.19694416177401
H	1.22087953965758	-1.40356272241228	-0.23910919489396
O	0.79242194700298	2.05639635682758	-0.11663989648942

Tl_2S* E = -742.8173203033 au
ZPE = 0.01257541 au
G_{corr} = -0.02156123 au

Tl	2.32160265462036	3.17671106305136	-0.10499765367589
H	3.75694290052738	4.05094701950840	-0.09909818476870
Tl	2.47524385212556	0.01377016961578	-0.21142513469292
H	1.21498251195092	-1.32512289588246	-0.23472401695869
S	0.28486021884398	2.15240067439187	-0.10619281816436

$[Tl_2S]^‡$ E = -742.8052262587 au
ZPE = 0.01233046 au
G_{corr} = -0.02071971 au
$\bar{\nu}$ = -219.74 cm^{-1}

Tl	2.83854317930362	2.77023859367375	-0.13304174354673
H	3.53119734539379	4.34770082823626	-0.09276187216677
Tl	1.90923277791185	0.19602890399181	-0.20512761104693
H	1.43457685140634	-1.45977378449605	-0.25643328510539
S	0.34008198405246	2.21451148927905	-0.06907329639473

Tl_2Se^* E = -2745.5247838618 au
ZPE = 0.01199750 au
G_{corr} = -0.02303566 au

Tl	3.59901867235313	2.46183996247376	-0.17315565734040
H	3.52077035938084	4.29613518830559	-0.12246888713446
Tl	1.43172582267630	0.16123021138204	-0.19615249944778
H	1.99101310412858	-1.43173527934416	-0.27529104768960
Se	0.43789049929521	2.34657542894531	-0.06512054465255

$[Tl_2Se]^‡$ E = -2745.5127914943 au
ZPE = 0.01183328 au
G_{corr} = -0.02224824 au
$\bar{\nu}$ = -193.05 cm^{-1}

Tl	3.04572056393465	2.71115765104588	-0.15659012495371
H	3.81501923296229	4.25465220629511	-0.12637243022497
Tl	2.07882830834793	0.14812324228206	-0.21667133447847
H	1.63706671453639	-1.51794487722952	-0.26134307069100
Se	0.40378363805264	2.23805728936891	-0.07121167591662

Tl_2Te^* E = -612.6719267238 au
ZPE = 0.01153824 au
G_{corr} = -0.02420759 au

Tl	3.61187438134301	2.44061654104324	-0.15378840998759
H	3.57179266461439	4.27535602673914	-0.09896078208203
Tl	1.45321441200617	0.13392216698914	-0.20296872473855
H	2.08920450507315	-1.44500125707071	-0.25792812101736
Te	0.25433249479738	2.42915203406173	-0.11854259843927

$[Tl_2Te]^‡$ E = -612.6598239630 au
ZPE = 0.01144301 au
G_{corr} = -0.02344796 au
$\bar{\nu}$ = -199.97 cm^{-1}

Tl	3.05099226254495	2.71327842275034	-0.13119432695210
H	3.90030299985543	4.21777791804717	-0.08487703086953

Tl	2.08596497756998	0.14241155690161	-0.20442000532689
H	1.73626948169469	-1.54887946169446	-0.25247062515556
Te	0.20688873616887	2.30945707575778	-0.15922664796069

Tl_2Po E = -582.3332379988 au
ZPE = 0.01127230 au
G_{corr} = -0.02513454 au

Tl	3.62349555562958	2.42050539952830	-0.15610830032670
H	3.58795930725935	4.25621087166115	-0.10111739614913
Tl	1.45603002686268	0.12508754985159	-0.20350404105464
H	2.11471543326660	-1.45455775142576	-0.25667685081874
Po	0.19821813481589	2.48679944214730	-0.11478204791561

C_2O E = -153.5907998927 au
ZPE = 0.05721936 au
G_{corr} = 0.03313659 au

C	-0.32154116244261	-0.11469124150117	0.16811474651976
H	-0.74683926427135	-0.49893981039342	1.09051857005839
H	0.76225235432793	-0.05005460684639	0.14438157577811
C	-1.06508771285172	-0.25518959101039	-1.08417054630843
H	-0.52679673356257	-0.29362106044761	-2.02656034868733
H	-2.03560332750774	-0.74251474147008	-1.08000820359659
O	-1.01036967293854	0.99857802665886	-0.40252543088217

C_2S E = -476.2254912340 au
ZPE = 0.05482397 au
G_{corr} = 0.02954157 au

C	-1.13973862678614	-0.50362065921846	0.33912635998046
H	-1.93476762962264	-1.19722287850735	0.58261485330845
H	-0.21346997430099	-0.66574303039183	0.87597172378170
C	-1.06605393483495	0.02066125348058	-1.04225481880719
H	-0.08876505737205	0.22177323041619	-1.46285160269118
H	-1.81012235794215	-0.30940709060704	-1.75631986528554
S	-1.65066506914108	1.23898834482792	0.18113105971331

C_2Se E = -2478.9206306312 au
ZPE = 0.05401992 au
G_{corr} = 0.02742897 au

C	-1.12639403723690	-0.53605418053374	0.32015901750967
H	-1.92901344139873	-1.21496204201429	0.57431180492198
H	-0.20495254470950	-0.68428029351786	0.86672984662089
C	-1.05499576194034	-0.01334267329716	-1.04783877188388
H	-0.08320427894931	0.20757885169397	-1.46799052400307
H	-1.80743907797796	-0.32254987266817	-1.76055288816861
Se	-1.69758349778726	1.36903937033725	0.23259922500303

C_2Te E = -346.0576244942 au
ZPE = 0.05321201 au
G_{corr} = 0.02636680 au

C	2.15916047774993	0.90443746980570	2.14546368150883
H	1.63564136722569	0.66424818028773	1.23077883241001
H	1.63564135259090	0.66424812044347	3.06014850428890
C	3.61113062437204	0.90447679258785	2.14546367245898
H	4.13466297241084	0.66431665193484	1.23077876661878
H	4.13466299797323	0.66431665239984	3.06014858323226
Te	2.88508840767738	2.99766940254057	2.14546364948223

C_2Po E = -315.7137880432 au
ZPE = 0.05288398 au
G_{corr} = 0.02455330 au

C	2.16759505479415	0.86793808081288	2.14546380725497
H	1.63565730972597	0.65759090505337	1.22881812054441
H	1.63565731649266	0.65758995274796	3.06210926678049
C	3.60269795506575	0.86797597538919	2.14546365275673
H	4.13464703116353	0.65765651644647	1.22881811854020
H	4.13464695173732	0.65765683819142	3.06210930367262
Po	2.88508658102060	3.09730500135870	2.14546342045056

Si_2O E = -655.7113555233 au
ZPE = 0.03778729 au
G_{corr} = 0.01112763 au

Si	-0.21935944835783	-0.04884242443336	1.82084491629874
H	-0.81429254043019	-1.23600571996207	2.47361946042535
H	-1.00385826798470	1.16303951785741	2.14536979994185
Si	1.68672518990055	-0.04880187246265	0.72067627932892
H	2.35965966075767	1.16303422380851	0.20293037304137
H	2.54923589099637	-1.23605117174018	0.53120959749124
O	0.09404432604327	-0.25099396539229	0.16270824703501

Si_2S E = -978.3210232946 au
ZPE = 0.03584811 au
G_{corr} = 0.00798180 au

Si	-0.25976625370716	0.02328065668147	1.80123183102251
H	-0.88016301805564	-1.21708150844338	2.31148431260302
H	-0.75839624622103	1.21308221933036	2.52193822568607
Si	1.68998538762144	0.02329868376662	0.67655819772372
H	2.56318086386578	1.21324450946929	0.60460488278968
H	2.44197825676225	-1.21695498382477	0.39372768179526
S	-0.20493132896097	0.20823881341153	-0.35592973094409

Si₂Se E = -2981.0159295831 au
ZPE = 0.03511244 au
G_{corr} = 0.00600624 au

Si	-0.26238449271880	0.02290980757157	1.80800121712462
H	-0.85532009026310	-1.22182996249581	2.33929619805681
H	-0.73143296243035	1.21046924179071	2.55171846463364
Si	1.69718804150963	0.02289375171884	0.67804806990185
H	2.57555302429549	1.21059032352516	0.64264435795228
H	2.45381668886835	-1.22163675175893	0.42923108290926
Se	-0.28553254795654	0.22371198003958	-0.49532398990229

Si₂Te E = -848.1486404424 au
ZPE = 0.03448170 au
G_{corr} = 0.00446685 au

Si	-0.26549102109446	0.02223866074695	1.81747374001509
H	-0.81531320280380	-1.22916017762632	2.37947876635344
H	-0.69256362664223	1.20637008484615	2.59180951479767
Si	1.70688191816013	0.02228803287139	0.68008488359836
H	2.59092866147310	1.20649405327720	0.69636601835581
H	2.46857365523917	-1.22893718107380	0.48400465681244
Te	-0.40112872302723	0.24781491734956	-0.69560217925664

Si₂Po E = -817.8036359699 au
ZPE = 0.03415458 au
G_{corr} = 0.00333559 au

Si	-0.26334344693691	0.02161666701771	1.82327005320573
H	-0.79822856631721	-1.23187646315166	2.39578642687574
H	-0.67573223853722	1.20443628451984	2.60809530160171
Si	1.71058007607081	0.02173409482976	0.68494470564501
H	2.59666731536649	1.20446304877418	0.71900579170555
H	2.47406865451280	-1.23151104055719	0.50684692077034
Po	-0.45212413285409	0.25824579895849	-0.78433379912792

Ge₂O E = -4229.3281401637 au
ZPE = 0.03401660 au
G_{corr} = 0.00488403 au

Ge	-0.26104108596995	-0.04451910099248	1.87676528694163
H	-0.86428445743128	-1.29936400076138	2.52943313305253
H	-1.06386431758756	1.23071496859117	2.18313728506841
Ge	1.75590180875183	-0.04461205060185	0.71160536967643
H	2.42218938423502	1.23103825625577	0.17021523104491
H	2.62230181233421	-1.29970956809467	0.51599344619461
O	0.04095166659288	-0.26816991672118	0.07020892158397

Ge_2S E = -4551.9636837971 au
ZPE = 0.03263327 au
G_{corr} = 0.00236934 au

Ge	-0.29630326197309	0.02038803522928	1.85110279737258
H	-0.94669458082824	-1.27715927589501	2.34758360440008
H	-0.81675846251486	1.27272807508430	2.56803450484023
Ge	1.75123283017546	0.02045774127776	0.66861455189576
H	2.63217361476614	1.27295422147446	0.57745460977018
H	2.50659526118869	-1.27717111738058	0.35463273794075
S	-0.23835773950943	0.21491071060092	-0.41380740554342

Ge_2Se E = -6554.6685309473 au
ZPE = 0.03196088 au
G_{corr} = 0.00051208 au

Ge	-0.29896416655647	0.02027733932812	1.85462136627002
H	-0.92210756304289	-1.28076936204556	2.37528961242369
H	-0.78907534367455	1.26963399996153	2.59726538343838
Ge	1.75557915539722	0.02034390683924	0.66817255015392
H	2.64381967542372	1.26972169287159	0.61598300899773
H	2.51855838220470	-1.28065548941138	0.38996011042178
Se	-0.31592247844707	0.22855630284759	-0.54767663102935

Ge_2Te E = -4421.8076353376 au
ZPE = 0.03145351 au
G_{corr} = -0.00087917 au

Ge	-0.30364954840814	0.02065374364274	1.85821396262200
H	-0.88080164127192	-1.28614228676782	2.41696768882104
H	-0.75228130232403	1.26241567918490	2.63902585398152
Ge	1.76106559270423	0.02097885682355	0.66432115773211
H	2.66156056537805	1.26270129919756	0.66920424353923
H	2.53372434817413	-1.28620584000165	0.44715286593178
Te	-0.42773035294767	0.25270693831185	-0.74127037195152

Ge_2Po E = -4391.4654318811 au
ZPE = 0.03116854 au
G_{corr} = -0.00194291 au

Ge	-0.30189488189337	0.02045489151428	1.86159795567672
H	-0.86389102704538	-1.28779965468813	2.43391785332986
H	-0.73643919136417	1.25905488182453	2.65621789989614
Ge	1.76305864929398	0.02128830157511	0.66550728989637
H	2.66881921174277	1.25944042163541	0.69220634883498
H	2.53958600309289	-1.28789407139134	0.47102682634114
Po	-0.47735110252207	0.26256361992127	-0.82685877329905

Sn_2O E = -505.2141415239 au

ZPE = 0.02898383 au
G_{corr} = -0.00223520 au

Sn	-0.38651509914498	-0.03639286773123	2.00942350017298
H	-1.10917576400250	-1.43928612114534	2.67886884621069
H	-1.33063863522761	1.36556978229988	2.29511719978422
Sn	1.93340785082985	-0.03619081482583	0.67009856946083
H	2.65276200724039	1.36546086183447	-0.00555622281108
H	2.87439375530224	-1.43914858654743	0.37860036648658
O	0.01792069592776	-0.27463366620913	0.03080641425827

Sn_2S E = -827.8656715140 au
ZPE = 0.02794683 au
G_{corr} = -0.00440657 au

Sn	-0.42136194450544	0.01579669354714	1.97688593088848
H	-1.20977880482894	-1.41448965786743	2.47943683001205
H	-1.06726121717457	1.41076992654287	2.72309533290933
Sn	1.92289955673518	0.01584017460507	0.62472582852619
H	2.89156591346595	1.41103054961690	0.43752476139691
H	2.75202760846953	-1.41430896856197	0.19221375530483
S	-0.27620345085704	0.22246967250856	-0.48026703836163

Sn_2Se E = -2830.5697911396 au
ZPE = 0.02735489 au
G_{corr} = -0.00614486 au

Sn	-0.42861930752855	0.01671535883475	1.97405925515130
H	-1.18487807881386	-1.41756590327543	2.51288193558542
H	-1.04166866520535	1.40510247721666	2.75898867043952
Sn	1.92424796241804	0.01656150124844	0.61820512674238
H	2.90910146354022	1.40562569868726	0.47708722892410
H	2.76793424140481	-1.41754140023456	0.22993617604800
Se	-0.35422995451065	0.23821065791401	-0.61754299221454

Sn_2Te E = -697.7130419891 au
ZPE = 0.02694178 au
G_{corr} = -0.00740015 au

Sn	-0.43919246296897	0.01772975643251	1.97008458396127
H	-1.14375388576747	-1.42230905534294	2.56220848720710
H	-1.00171849464268	1.39698841942340	2.80794962037077
Sn	1.92578651618829	0.01769606766665	0.60614946805949
H	2.93242680741339	1.39710224070402	0.53650300481702
H	2.79111561861666	-1.42200506526759	0.29101061417474
Te	-0.47277643753459	0.26190602677506	-0.82029037791422

Sn_2Po E = -667.3725824491 au
ZPE = 0.02669797 au

G_{corr} = -0.00837594 au

Sn	-0.44175306735727	0.01791713205213	1.96900074047521
H	-1.12578521255468	-1.42411547880810	2.58134455172612
H	-0.98406777403454	1.39355422755273	2.82685428101847
Sn	1.92623931447166	0.01792372428249	0.60440909700394
H	2.94022360220023	1.39362133779975	0.56092045227970
H	2.79871020635152	-1.42365169948079	0.31590818059311
Po	-0.52167940777229	0.27185914699290	-0.90482190242037

Pb_2O E = -758.1898519337 au
ZPE = 0.02220533 au
G_{corr} = -0.00412571 au

Pb	-0.57204605656255	-0.06184595934916	1.92478679361400
H	-1.12003963959525	-1.50895821028574	2.77685341869459
H	-1.35489876382411	1.45886447220177	2.36649167311545
Pb	2.07135740084132	-0.00057364419726	0.85208133103994
H	2.69480685236624	1.37138920357042	-0.11092158845278
H	2.92047329978317	-1.46727028650094	0.28120042300500
O	0.01250171791630	-0.28622698776370	-0.03313337745375

Pb_2S E = -1080.8237675125 au
ZPE = 0.02089327 au
G_{corr} = -0.00660420 au

Pb	-0.69539632438487	-0.08199642715158	1.84872575107262
H	-0.99991383465804	-1.46042123171005	2.90872119724272
H	-1.23622130527741	1.46583831291041	2.50283190197243
Pb	2.09296528375544	0.01634728143282	0.95507579442895
H	2.75379618230543	1.36694442238321	-0.01526153815116
H	2.97399130160867	-1.43031560802654	0.37685438748174
S	-0.23706649242411	-0.37101816216289	-0.51958882048485

Pb_2Se E = -3083.5221623218 au
ZPE = 0.02030780 au
G_{corr} = -0.00841403 au

Pb	-0.72260699289377	-0.08530560622173	1.84266372221517
H	-0.97904740523418	-1.44473557527829	2.94253403581732
H	-1.19853655391653	1.46838776859173	2.53821340779664
Pb	2.09777252113566	0.01779038178174	0.98061118570074
H	2.76373407549263	1.37087727603836	0.01460767009387
H	2.98619050258014	-1.42085946751701	0.39140723312884
Se	-0.29535133623883	-0.40077618971942	-0.65267858119012

Pb_2Te E = -950.6627366250 au
ZPE = 0.01990559 au
G_{corr} = -0.00968353 au

Pb	-0.76750007194914	-0.08958513934374	1.83326277344246
H	-0.93304388610484	-1.42070395550311	2.99023185875890
H	-1.15745973485839	1.46350013460106	2.59070907154238
Pb	2.10952314768018	0.02638475725578	1.02966078557497
H	2.77583137451820	1.36756070921318	0.04182445922927
H	2.99508203997213	-1.41086023058142	0.42262870881372
Te	-0.37027805833303	-0.43091768796638	-0.85095898379924

Pb_2Po E = -920.3212755485 au
ZPE = 0.01969272 au
G_{corr} = -0.01067449 au

Pb	-0.78771799596289	-0.09201693496750	1.82857230011158
H	-0.91366869285037	-1.40875391481020	3.01126169549346
H	-1.13831371063910	1.46003426562332	2.61381257669655
Pb	2.11397585470445	0.03109634365892	1.05635649031961
H	2.77759329985553	1.36395036835353	0.05087766767129
H	2.99463085850418	-1.40520162849836	0.43220560007625
Po	-0.39434480268669	-0.44372991168433	-0.93572765680628

N_2O E = -185.5571719213 au
ZPE = 0.03360635 au
G_{corr} = 0.00964394 au

N	0.05298164430198	0.75987836153245	0.07042470028866
H	-0.26306474436111	0.75022063088847	-0.90545295649706
N	1.48555333447367	0.68543624326147	-0.03979634739881
H	1.78475789723148	0.41619944223404	0.90374622033193
O	0.84495896370247	1.96819161158326	0.15972737067003

N_2S E = -508.2325786683 au
ZPE = 0.03178241 au
G_{corr} = 0.00666837 au

N	0.04086928382066	0.70747694164684	0.05731894735818
H	-0.27205499157532	0.65322866024911	-0.91407445205543
N	1.49127654878042	0.63061810330143	-0.03909825966377
H	1.78184129802980	0.31928586841468	0.88996194200043
S	0.86325495629293	2.26931671588763	0.19454080975534

N_2Se E = -2510.9323088167 au
ZPE = 0.03112661 au
G_{corr} = 0.00471209 au

N	0.05023800563507	0.65569531131048	0.05435361706886
H	-0.27023114553712	0.63619906220155	-0.91619510045298
N	1.47554336353773	0.58094728021744	-0.04785359207421
H	1.77812591920204	0.30265581946027	0.88810377194413

Se	0.87151095251078	2.40442881630994	0.21024029090894

N_2Te E = -378.0795791612 au
ZPE = 0.03053024 au
G_{corr} = 0.00323650 au

N	0.05841819448054	0.60376941194146	0.04887074258194
H	-0.27070213991354	0.60268942147966	-0.91951910551636
N	1.46119969246424	0.53031792621397	-0.05426630573349
H	1.77454641706514	0.26943995427429	0.88370996814106
Te	0.88172493125212	2.57370957559030	0.22985368792160

N_2Po E = -347.7389130003 au
ZPE = 0.03030340 au
G_{corr} = 0.00217774 au

N	0.07119769359152	0.56050287500644	0.04574418855509
H	-0.26802517624622	0.59531142821948	-0.91961986803669
N	1.44340225388737	0.48883274210999	-0.06100277575099
H	1.77102275208800	0.26265729177154	0.88217931823456
Po	0.88758957202783	2.67262195239223	0.24134812439278

P_2O E = -758.1898519337 au
ZPE = 0.02220533 au
G_{corr} = -0.00412571 au

P	-0.30079582785032	0.77709892019132	0.15097099243085
H	-0.60741035721103	0.77284739133402	-1.24796573989502
P	1.84009275623702	0.67842901879358	-0.11899620649348
H	2.12460159134456	0.31842794447832	1.23780007118194
O	0.84869893282826	2.03312301470245	0.16683987017046

P_2S E = -1080.8237675125 au
ZPE = 0.02089327 au
G_{corr} = -0.00660420 au

P	-0.33044934598947	0.61303769097199	0.17681598091583
H	-0.58537620853423	0.66354143034767	-1.22477858377348
P	1.86406248650637	0.49236867666632	-0.07469041255206
H	2.09493078145440	0.10308561332677	1.27694247373436
S	0.89667845217752	2.36164307168393	0.31745968690450

P_2Se E = -3083.5221623218 au
ZPE = 0.02030780 au
G_{corr} = -0.00841403 au

P	-0.33604213453637	0.57086397207281	0.17338794090110
H	-0.58369874484605	0.63985008253849	-1.22682695531532

P	1.86388014978212	0.45120247073095	-0.08316621422964
H	2.09001068619591	0.07919734849451	1.27235491145695
Se	0.90569620901897	2.49256260915992	0.33599946241607

P_2Te E = -950.6627366250 au
ZPE = 0.01990559 au
G_{corr} = -0.00968353 au

P	-0.34259878192702	0.51527195859225	0.16719095701001
H	-0.58418644482022	0.60525669108487	-1.23142757689402
P	1.86253116634022	0.39551083348788	-0.09372197232888
H	2.08515556943193	0.04607342823791	1.26677720929567
Te	0.91894465658967	2.67156357159377	0.36293052814638

P_2Po E = -920.3212755485 au
ZPE = 0.01969272 au
G_{corr} = -0.01067449 au

P	-0.34224730647694	0.48745826867127	0.16456189858812
H	-0.58350222889882	0.59046141062124	-1.23291074671702
P	1.85904602963452	0.36854178157661	-0.09928435993760
H	2.08159682910118	0.03169026695730	1.26412798200956
Po	0.92495284225464	2.75552475517027	0.37525437128611

As_2O E = -4545.8868139274 au
ZPE = 0.01920190 au
G_{corr} = -0.00951551 au

As	1.14210842104164	0.53659818881355	-0.30765643164222
H	1.64886472993092	0.50238462587496	1.14928341852607
As	-1.16452130667474	0.54987607341267	0.34531253420754
H	-1.65811289125183	0.90897264036103	-1.07158828029421
O	-0.06816893991389	-0.85346570892556	-0.15349347529405

As_2S E = -4868.5352995945 au
ZPE = 0.01808118 au
G_{corr} = -0.01174211 au

As	1.17188272399867	0.63796281792052	-0.30165781007194
H	1.64943822782317	0.60084869127453	1.15753099160869
As	-1.18721896680904	0.65086566170393	0.36419008616164
H	-1.64985566399324	1.00708160092805	-1.05646198420205
S	-0.08407630788746	-1.25239295229038	-0.20174351799321

As_2Se E = -6871.2375123305 au
ZPE = 0.01752025 au
G_{corr} = -0.01346028 au

As	1.17721158330888	0.67519184862011	-0.29922603897390
H	1.64793280043646	0.62463264018350	1.15979440666708
As	-1.18955844710381	0.68890744385092	0.37184707484176
H	-1.64544486169084	1.03167193192048	-1.05214861031735
Se	-0.08997106181860	-1.37603804503835	-0.21840906671444

As_2Te E = -4738.3815096137 au
ZPE = 0.01718596 au
G_{corr} = -0.01464180 au

As	1.18451091114388	0.72837482282585	-0.29571936366490
H	1.64560722050219	0.65786648950910	1.16351842576398
As	-1.19070838610104	0.74263887976169	0.38160984931630
H	-1.64148949647383	1.06623010505199	-1.04658892404593
Te	-0.09775023593910	-1.55074447761196	-0.24096222186630

As_2Po E = -4708.0416763036 au
ZPE = 0.01699738 au
G_{corr} = -0.01558373 au

As	1.18510500646052	0.75280101133335	-0.29434479625165
H	1.64219596503854	0.67442692924319	1.16534330655392
As	-1.18682094607946	0.76753599852534	0.38609122526361
H	-1.63891967727029	1.08148001530660	-1.04365766648456
Po	-0.10139033501722	-1.63187813487182	-0.25157430357818

Sb_2O E = -555.9725001344 au
ZPE = 0.01641562 au
G_{corr} = -0.01406610 au

Sb	-0.59511009905103	0.64259543767858	0.25427876148205
H	-0.85698165345202	0.75949443581022	-1.45386562415595
Sb	2.14890129967618	0.48114989559827	-0.05476166350550
H	2.38342491805494	0.03705758319415	1.60287268589216
O	0.88864997295099	2.01994270808394	0.32571474979179

Sb_2S E = -878.6248782360 au
ZPE = 0.01541782 au
G_{corr} = -0.01616116 au

Sb	-0.63256606181170	0.54227916682327	0.24086696282799
H	-0.86109701693234	0.64806700752870	-1.46695559718746
Sb	2.16948827745119	0.37610809901177	-0.07314940180225
H	2.37242127267997	-0.07229945278287	1.58148668098142
S	0.92063796679195	2.44608523978429	0.39199026468485

Sb_2Se E = -2881.3296780380 au
ZPE = 0.01488669 au

G_{corr} = -0.01782414 au

Sb	-0.64083486877967	0.50688388834195	0.23706224780646
H	-0.86144041512126	0.62282995788576	-1.46926450805217
Sb	2.17349175693942	0.34165746906387	-0.07930681905014
H	2.36861944993934	-0.09934598873620	1.57675286127361
Se	0.92904851520123	2.56821473380978	0.40899512752679

Sb₂Te E = -748.4747133544 au
ZPE = 0.01460454 au
G_{corr} = -0.01892635 au

Sb	-0.65167749417538	0.45533694377597	0.23117600311212
H	-0.86212151215504	0.58402926798535	-1.47373235245183
Sb	2.17716098537844	0.29005597603093	-0.08994357645568
H	2.36158407051504	-0.13535236326680	1.56915104508397
Te	0.94393838861600	2.74617023583970	0.43758779021597

Sb_2Po E = -718.1355257258 au
ZPE = 0.01443726 au
G_{corr} = -0.01980665 au

Sb	-0.65357574529974	0.43575612160405	0.23092463105596
H	-0.86189610307768	0.56750684157507	-1.47386328383731
Sb	2.17354194797407	0.26913861426617	-0.09693198295136
H	2.35944027293502	-0.15909186146074	1.56072895710277
Po	0.95137406564738	2.82693034438061	0.45338058813448

Bi_2O E = -504.4874544750 au
ZPE = 0.01494339 au
G_{corr} = -0.01722131 au

Bi	-0.67079319169324	0.62419593923895	0.26210768231207
H	-0.93343536697753	0.75710699349240	-1.53128591318573
Bi	2.22573639089113	0.47162129927846	-0.06756160799581
H	2.46195640193524	0.01626305136816	1.67596414637760
O	0.88542020402346	2.07105277698718	0.33501460199643

Bi_2S E = -827.1483410196 au
ZPE = 0.01403570 au
G_{corr} = -0.01919254 au

Bi	-0.70875446891964	0.52946587273105	0.24566008607137
H	-0.94154214884031	0.65297196558789	-1.54645100051314
Bi	2.24683011431823	0.36675884583035	-0.08374244505789
H	2.45482515202111	-0.10121726435009	1.65372389050768
S	0.91752578959968	2.49226064056596	0.40504837849653

Bi_2Se E = -2829.8555750678 au
ZPE = 0.01352297 au
G_{corr} = -0.02080041 au

Bi	-0.71764495085165	0.49955318333898	0.24235943438032
H	-0.94352363666548	0.62702120623054	-1.54837223305870
Bi	2.25058851168281	0.33140361371653	-0.08879483560242
H	2.45040556378660	-0.12971618639937	1.64981018876255
Se	0.92905895022677	2.61197824347848	0.41923635502279

Bi_2Te E = -697.0025214175 au
ZPE = 0.01326778 au
G_{corr} = -0.02182119 au

Bi	-0.73029674819952	0.44968269713860	0.23505198414614
H	-0.94198359529276	0.59016594384902	-1.55426163078316
Bi	2.25531555257524	0.28036067828101	-0.09802668179032
H	2.44345049450246	-0.16437940128573	1.64357291240543
Te	0.94239873459363	2.78441014238226	0.44790232552646

Bi_2Po E = -666.6640945537 au
ZPE = 0.01310636 au
G_{corr} = -0.02263564 au

Bi	-0.73156580558532	0.43158048655487	0.23391185633290
H	-0.93965613828490	0.56700954163711	-1.55594629931698
Bi	2.25314792616934	0.26287461163362	-0.10231374234441
H	2.43882328632104	-0.18568672842106	1.63820070799266
Po	0.94813516955889	2.86446214896062	0.46038638684038

O_3 E = -225.1534464743 au
ZPE = 0.00663501 au
G_{corr} = -0.01659595 au

O	0.09386091012430	0.06440724156109	0.03526965149479
O	0.09393865576692	0.06449755394045	1.46580442234933
O	1.32384627919291	-0.08510885777805	0.75047955747799

O_2S E = -547.8788074030 au
ZPE = 0.00553237 au
G_{corr} = -0.01878405 au

O	-0.05931972724329	0.03724290460917	0.01143813138580
O	-0.05923924395274	0.03733567196199	1.48965584070930
S	1.41729965119990	-0.25227254971263	0.75048553128920

O_2Se E = -2550.5800759776 au
ZPE = 0.00496045 au

G_{corr} = -0.02128452 au

O	-0.13615455402934	0.03218237662246	0.01590269354031
O	-0.13607445039589	0.03227487214892	1.48520002247353
Se	1.50147161622709	-0.30802517482530	0.75048505559012

O_2Te E = -417.7367814831 au
ZPE = 0.00464238 au
G_{corr} = -0.02177481 au

O	-0.23689719065518	0.01378224814689	0.01632249439976
O	-0.23681870982941	0.01387433684603	1.48479418995780
Te	1.57837790154835	-0.37992927756519	0.75048513480348

O_2Po E = -387.3955447094 au
ZPE = 0.00444081 au
G_{corr} = -0.02276021 au

O	-0.27514425380558	0.02207936231409	0.02483877704673
O	-0.27506658299320	0.02217036136681	1.47628095580178
Po	1.65487283786254	-0.39652241625318	0.75048208631253

S_2O E = -870.5754149811 au
ZPE = 0.00443801 au
G_{corr} = -0.02096907 au

S	0.00918194724687	0.02820332912301	-0.27286566284292
S	0.00929089628468	0.02830325937099	1.77394612822893
O	1.38407193985983	-0.04463671082694	0.75047072384674

S_3 E = -1193.2492076462 au
ZPE = 0.00343796 au
G_{corr} = -0.02373649 au

S	0.42930198475519	1.74609403670489	-0.42347371948631
S	2.03975597921129	1.79486032676776	0.91045801146058
S	1.10312864136450	-0.02240077628928	0.46737524022945

S_2Se E = -3195.9535613070 au
ZPE = 0.00293594 au
G_{corr} = -0.02544566 au

S	0.43639851834134	1.80951956202442	-0.44009900949792
S	2.05299253061865	1.85854392267431	0.89795530560267
Se	1.12626431384302	-0.12495943167104	0.44393161152705

S_2Te E = -1063.1058691443 au
ZPE = 0.00269361 au

G_{corr} = -0.02651302 au

S	0.46291073487864	1.91092824442597	-0.48348844297147
S	2.08700652228559	1.96017051077753	0.86079809738763
Te	1.15620393873446	-0.23181181212266	0.41171648085451

S_2Po E = -1032.7673804856 au
ZPE = 0.00255998 au
G_{corr} = -0.02741151 au

S	0.46563736047062	1.94676268146830	-0.48654134421393
S	2.08824838901012	1.99606998506196	0.85649989561808
Po	1.15223544641795	-0.30354572344943	0.41906758386652

Se_2O E = -4875.9837208330 au
ZPE = 0.00340236 au
G_{corr} = -0.02508844 au

Se	-0.02563000800796	0.02294445137069	-0.41063921660988
Se	-0.02551358371618	0.02304878759282	1.91172707067133
O	1.42913923704733	-0.04513709257167	0.75047663164094

Se_2S E = -5198.6589948880 au
ZPE = 0.00242918 au
G_{corr} = -0.02712420 au

Se	0.32410173873635	1.77403413874952	-0.51639974153404
Se	2.14937140855387	1.82900467113621	0.99551874881331
S	1.09871345804076	-0.08448522270236	0.47524052492445

Se_3 E = -7201.3656390025 au
ZPE = 0.00192089 au
G_{corr} = -0.02876759 au

Se	1.11650146581180	-0.17153050522153	0.45627901292439
Se	0.33741911950935	1.84294053307319	-0.54239916996697
Se	2.16941906524477	1.89795192281299	0.97602649015876

Se_2Te E = -5068.5199117309 au
ZPE = 0.00169908 au
G_{corr} = -0.02979647 au

Se	0.36331171195902	1.94469913368915	-0.58656866846225
Se	2.20501968783985	2.00004842697964	0.93844810614205
Te	1.14658579919313	-0.27783519535960	0.42483522675583

Se_2Po E = -5038.1829620023 au
ZPE = 0.00157634 au

G_{corr} = -0.03065857 au

Se	0.36561872620140	1.97887791647548	-0.59016820027781
Se	2.20679996621216	2.03400705747238	0.93444541843179
Po	1.14249850657844	-0.34597260863867	0.43243744628165

Te_2O E = -610.2953458128 au
ZPE = 0.00289867 au
G_{corr} = -0.02719682 au

Te	-0.06426641203617	0.01197367915610	-0.59366941504196
Te	-0.06412550582842	0.01210378340491	2.09476066250774
O	1.46008419148663	-0.03495546329581	0.75046503434386

Te_2S E = -932.9650737083 au
ZPE = 0.00197605 au
G_{corr} = -0.02920423 au

Te	0.19451472456048	1.82063901723550	-0.65247802976955
Te	2.30482842334383	1.88405390178672	1.09645327657721
S	1.08574995145189	-0.14744403331018	0.49243927927073

Te_2Se E = -2935.6735438692 au
ZPE = 0.00148470 au
G_{corr} = -0.03081396 au

Te	0.20681614782640	1.88996220699355	-0.67963338733358
Te	2.32568007942473	1.95301838740302	1.07794815331612
Se	1.10294061588927	-0.23430196777107	0.47429857253021

Te_3 E = -802.8266806001 au
ZPE = 0.00128055 au
G_{corr} = -0.03180855 au

Te	1.13175745592394	-0.34663922500830	0.44319616226101
Te	0.23214838878548	1.99549567146352	-0.72689877135283
Te	2.36173827372392	2.05723590932320	1.04161382011001

Te_2Po E = -772.4901840482 au
ZPE = 0.00116472 au
G_{corr} = -0.03263222 au

Te	1.10876206428218	-0.36410938538657	0.42710117886872
Te	0.20802362903137	1.97886947999827	-0.74408823606187
Po	2.40885842511979	2.09133226116673	1.07489826821134

Po_2O E = -549.6173413648 au
ZPE = 0.00260248 au

G_{corr} = -0.02831853 au

Po	-0.08816638110864	0.00956101093368	-0.67786612161052
Po	-0.08801418514943	0.00969971298046	2.17895754298204
O	1.49447222568337	-0.03768742816995	0.75046422026096

Po₂S E = -872.2901966447 au
ZPE = 0.00173061 au
G_{corr} = -0.03091047 au

Po	0.13480164913250	1.84312805015715	-0.71387745389868
Po	2.37575633029091	1.91081370241190	1.14278779402967
S	1.08021940317010	-0.18122703877345	0.50040345937265

Po₂Se E = -2875.0003230193 au
ZPE = 0.00124387 au
G_{corr} = -0.03248878 au

Po	0.14318290496387	1.90711654995130	-0.73605286455513
Po	2.39201384544986	1.97335992051969	1.12946523772542
Se	1.10024009272666	-0.27179784384549	0.47920096534246

Po₂Te E = -742.1538844912 au
ZPE = 0.00104905 au
G_{corr} = -0.03343113 au

C	2.15916047774993	0.90443746980570	2.14546368150883
H	1.63564136722569	0.66424818028773	1.23077883241001
H	1.63564135259090	0.66424812044347	3.06014850428890
C	3.61113062437204	0.90447679258785	2.14546367245898
H	4.13466297241084	0.66431665193484	1.23077876661878
H	4.13466299797323	0.66431665239984	3.06014858323226
Te	2.88508840767738	2.99766940254057	2.14546364948223

Po₃ E = -711.8175817323 au
ZPE = 0.00093016 au
G_{corr} = -0.03355553 au

Po	-0.01386815305569	-0.00756496932502	0.00000000000000
Po	2.92757966042897	-0.00756501615289	0.00000000000000
Po	1.45685571262672	2.53847814547791	0.00000000000000

AlCGe E = -2358.8915507058 au
ZPE = 0.04584209 au
G_{corr} = 0.01688894 au

Al	0.00136452985174	0.00811350413886	-0.01130338182522
H	0.25115651523616	-0.01548596211865	1.54628367767833
C	0.84333905564418	0.00297469597255	-1.75416695212991

H	1.32347030843778	-0.89667041360220	-2.14048655556131
H	1.37531105113627	0.88105017237025	-2.12171365703288
Ge	-1.20317838783012	0.06562454647936	-2.05971603887332
H	-1.71744311069916	-1.16609017629030	-2.83181565448293
H	-1.64291937177683	1.34423148305014	-2.80129125777274

CGeSe E = -4516.7826891892 au
ZPE = 0.04227701 au
G_{corr} = 0.01326689 au

C	-0.02089474017336	-0.06930556173320	-0.01600845606978
H	-0.08731545419590	0.00819447976828	1.06156918734338
H	0.98491276439405	0.01147692438732	-0.40761696653658
Ge	-1.29598260318606	-1.18482200756765	-0.94833395526064
H	-2.38673129067339	-1.89351613090703	-0.14485494325265
H	-0.86290098690150	-1.88841475972198	-2.23479773873582
Se	-1.33107714926383	1.15840824577425	-0.96919640748791

CNS E = -492.2365856621 au
ZPE = 0.04336880 au
G_{corr} = 0.01817638 au

C	0.00232715514336	-0.03901260292214	0.03739872657276
H	-0.02453184109154	-0.04317360700226	1.12064096897771
H	0.99056234066963	0.02049847184690	-0.40678877950418
N	-0.99921593785732	-0.86005514751940	-0.59152457588429
H	-0.65244127281039	-1.17982808584863	-1.49504888732795
S	-1.29311496405374	0.91274262144555	-0.76587717283405

CPSe E = -2781.2147080661 au
ZPE = 0.03670738 au
G_{corr} = 0.00905941 au

C	-0.01796969385619	-0.06891501598749	-0.02143429979475
H	-0.00640499025884	0.08023587865766	1.05071105661775
H	0.96002796196044	-0.14214143355515	-0.47898901112255
P	-1.38035720854410	-1.10756660126229	-0.67600052603716
H	-0.71663641962134	-1.48893194897974	-1.87679855772200
Se	-1.26373044967997	1.14904607112699	-0.96549286194129

SiPS E = -1029.5753227813 au
ZPE = 0.02834590 au
G_{corr} = 0.00067245 au

S	-0.05216000751901	-0.01113431587369	0.07187709079923
Si	-0.00695528313616	-0.06902488785419	2.18152135908533
H	1.31184653160082	0.02209747043894	2.83421439581108
H	-1.05591788097895	0.67400745021025	2.90521348194597
P	-0.51393151775748	-1.92653614105310	1.07132342822245

H	-1.92129573220922	-1.73441919586820	1.20461462413593

SnAsTe E = -2718.0463381582 au
ZPE = 0.02202440 au
G_{corr} = -0.01101372 au

Te	0.01921157908902	-0.01965680987199	0.16784945276139
As	-0.27012104302584	-0.26009268101649	2.84282983615081
H	1.02403798725931	0.48566653447517	3.18608960388216
Sn	-1.24449445283304	1.86298189285740	1.68682616367082
H	-0.46712131041686	3.37155019474090	1.85184067480023
H	-2.93482701007258	1.97711213881500	1.49694521873459
